# The *Azospirillum brasilense* Core Chemotaxis Proteins CheA1 and CheA4 Link Chemotaxis Signaling with Nitrogen Metabolism

**DOI:** 10.1128/mSystems.01354-20

**Published:** 2021-02-16

**Authors:** Elena E. Ganusova, Lam T. Vo, Paul E. Abraham, Lindsey O’Neal Yoder, Robert L. Hettich, Gladys Alexandre

**Affiliations:** a Department of Biochemistry and Cellular and Molecular Biology, University of Tennessee, Knoxville, Tennessee, USA; b Oak Ridge National Laboratory, Oak Ridge, Tennessee, USA; University of Vienna

**Keywords:** *Azospirillum*, chemotaxis, nitrate assimilation, nitrogen fixation, RpoN, metabolomics, nitrogen metabolism, proteomics

## Abstract

Bacterial chemotaxis affords motile bacteria the ability to navigate the environment to locate niches for growth and survival. At the molecular level, chemotaxis depends on chemoreceptor signaling arrays that interact with cytoplasmic proteins to control the direction of movement. In Azospirillum brasilense, chemotaxis is mediated by two distinct chemotaxis pathways: Che1 and Che4. Both Che1 and Che4 are critical in the *A. brasilense* free-living and plant-associated lifestyles. Here, we use whole-cell proteomics and metabolomics to characterize the role of chemotaxis in *A. brasilense* physiology. We found that mutants lacking CheA1 or CheA4 or both are affected in nonchemotaxis functions, including major changes in transcription, signaling transport, and cell metabolism. We identify specific effects of CheA1 and CheA4 on nitrogen metabolism, including nitrate assimilation and nitrogen fixation, that may depend, at least, on the transcriptional control of *rpoN*, which encodes RpoN, a global regulator of metabolism, including nitrogen. Consistent with proteomics, the abundance of several nitrogenous compounds (purines, pyrimidines, and amino acids) changed in the metabolomes of the chemotaxis mutants relative to the parental strain. Further, we uncover novel, and yet uncharacterized, layers of transcriptional and posttranscriptional control of nitrogen metabolism regulators. Together, our data reveal roles for CheA1 and CheA4 in linking chemotaxis and nitrogen metabolism, likely through control of global regulatory networks.

**IMPORTANCE** Bacterial chemotaxis is widespread in bacteria, increasing competitiveness in diverse environments and mediating associations with eukaryotic hosts ranging from commensal to beneficial and pathogenic. In most bacteria, chemotaxis signaling is tightly linked to energy metabolism, with this coupling occurring through the sensory input of several energy-sensing chemoreceptors. Here, we show that in *A. brasilense* the chemotaxis proteins have key roles in modulating nitrogen metabolism, including nitrate assimilation and nitrogen fixation, through novel and yet unknown regulations. These results are significant given that *A. brasilense* is a model bacterium for plant growth promotion and free-living nitrogen fixation and is used as a bio-inoculant for cereal crops. Chemotaxis signaling in *A. brasilense* thus links locomotor behaviors to nitrogen metabolism, allowing cells to continuously and reciprocally adjust metabolism and chemotaxis signaling as they navigate gradients.

## INTRODUCTION

**FIG 1:**
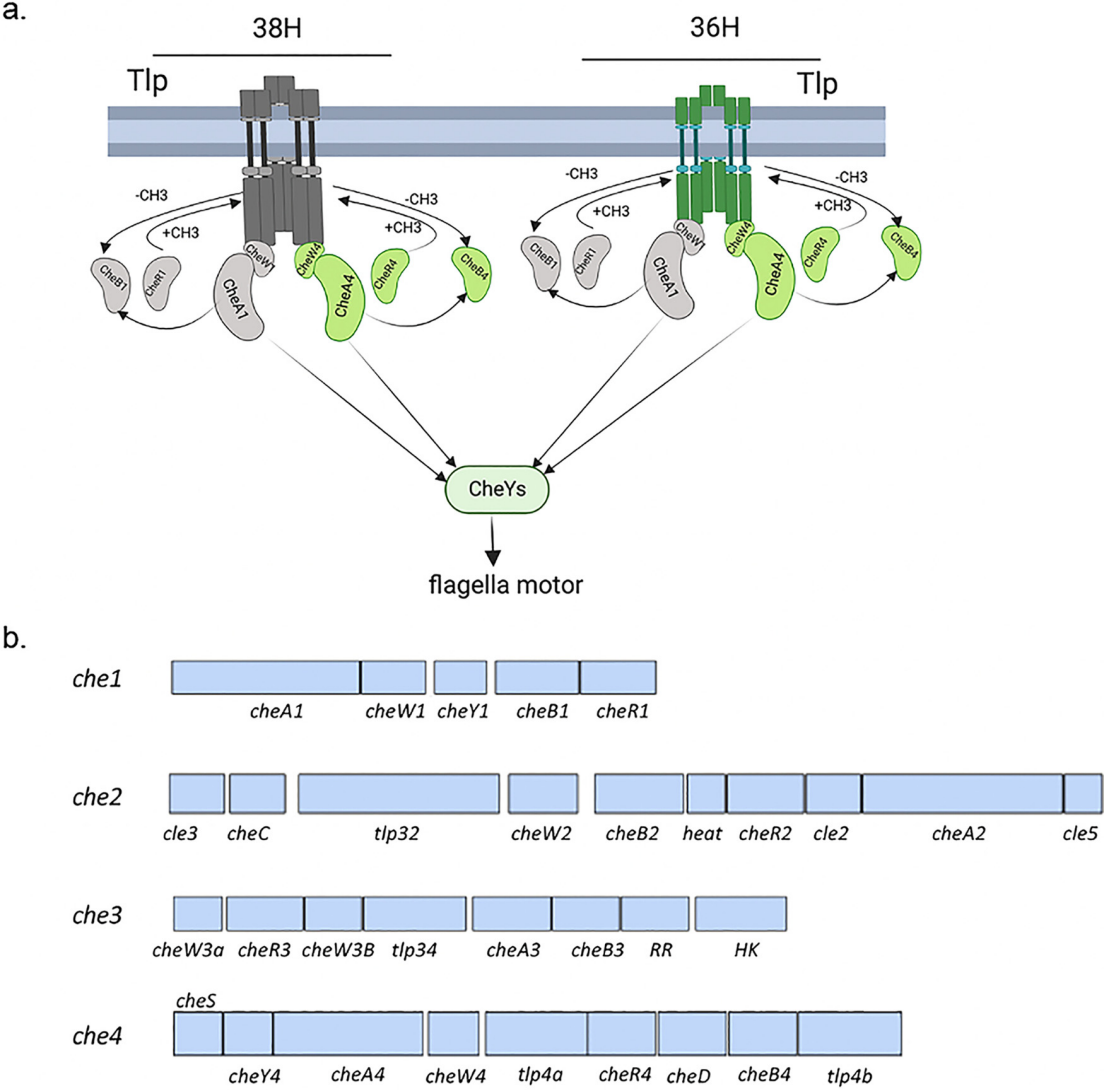
Chemotaxis signaling in *A. brasilense.* (a) Membrane-bound chemotaxis receptors (Tlps) are organized in two spatially distinct signaling arrays that assemble with chemoreceptors belonging to either the 38H (H, heptad, colored in gray) length class or the 36H length class (colored in green). For the comparison, 38H and 36H clusters are depicted with chemoreceptors of different lengths. CheA1 and CheA4, as well as CheW1 and CheW4 proteins, form mixed base plates that are required for the assembly of both chemoreceptor arrays. Signals (chemoattractant or repellent) received by Tlps are transduced through the array and modulate changes in their conformations and the autophosphorylation activities of CheA1 and/or CheA4, which ultimately affect the phosphorylation states of flagellar-motor switching response regulators (CheYs). The signaling activity of Tlps is also regulated by the combined activity of a chemoreceptor-specific methyltransferase (CheR1 and CheR4), which adds methyl groups to the chemoreceptors to keep them in an inactive state. These methyl groups are removed by the chemoreceptor-specific methylesterase, CheB1 and CheB4, whose activity depends upon phosphorylation from CheA1 and CheA4, respectively. Note that in *A. brasilense*, some chemoreceptors also possess c-di-GMP binding PilZ domains that are not represented here. (b) Chemotaxis and chemosensory pathways genomic organization within the *A. brasilense* genome. Boxes represent open reading frames and are drawn to scale. The chemotaxis genes within each cluster were either previously characterized or identified by homology searches, as detailed in reference 9 with modifications.

Chemotaxis is the directional movement of a motile organism toward an attractant (e.g., a source of food) or away from a repellent through modulation of the motility pattern. Movement of flagellated bacteria in a gradient comprises a sequence of smooth swimming runs interrupted by changes in the swimming direction (here, reversals) that bias the directional movement of swimming cells. Bacterial chemotaxis is mediated via dedicated signal transduction systems that involve chemoreceptors and two-component chemoreceptor-regulated phosphorylation pathways, which have been best described in Escherichia coli (1–3). The chemotaxis signal transduction is initiated by signal recognition of effectors by a cluster of chemoreceptors, which together modulate the autophosphorylation activity of a chemoreceptor-associated histidine kinase (CheA). Repellents stimulate CheA phosphorylation by ATP which subsequently donates phosphoryl group to its cognate response regulator, CheY. CheY∼P controls the direction of rotation of the flagella motors (4). Phospho-activated CheA also activates a chemoreceptor-specific methylesterase, CheB, which counteracts the activity of a constitutive chemoreceptor-specific methyltransferase, CheR, to control the magnitude of the stimulus response (5) (Fig. 1a). CheR adds methyl groups from *S*-adenosylmethionine to specific glutamate residues on the C-terminal domain of chemoreceptors. Upon activation by phosphotransfer from CheA, phosphorylated CheB removes the methyl groups in the form of methanol (6).

In *A. brasilense*, chemotaxis is controlled via two chemotaxis (Che) pathways, Che1 and Che4. The Che1 signaling output controls transient changes in swimming speed, while Che4 controls the frequency of swimming reversals. Mutants lacking Che1 pathway proteins, including CheA1, have reduced chemotaxis but are not null. Mutants lacking the Che4 pathway proteins, or lacking CheA4, are chemotaxis null (7–9). CheA1 and CheA4 proteins are central regulators of the chemotaxis response and together coordinate changes in the swimming motility pattern in response to attractants and repellents (7–11) and mediate recognition of specific regions of the root surface and root colonization (11). The exact function of two other chemosensory pathways encoded on the genome (*che2* and *che3*) is not known, but they are not predicted or needed for chemotaxis (Fig. 1b) (11). Che3 was suggested to contribute to flocculation (12) by an unknown mechanism.

The genome of *A. brasilense* encodes 51 predicted chemoreceptors, only 3 of which have been characterized: Tlp1 is a membrane-bound chemoreceptor that mediates taxis to organic acids and other energy taxis responses, such as aerotaxis, while AerC and Aer are soluble energy taxis transducers (13–15). In all *Bacteria* and *Archaea* studied to date for chemotaxis, chemoreceptors assemble as higher-order membrane-bound polar arrays with CheA and the coupling protein CheW forming large assemblies that are critical for signal propagation and amplification (1, 2, 16, 17). The cytoplasmic domains of chemoreceptors are comprised of repeated heptads (H stands for heptad) and chemoreceptors of the same heptad length class cluster together (17, 18). In *A. brasilense*, two membrane-bound chemoreceptor arrays are detected by cryo-EM of multiple cells of the wild type: an array comprised of 38H chemoreceptors and a second, spatially distinct array predicted to comprise 36H length class chemoreceptors. Both Che1 and Che4 cytoplasmic proteins (such as CheA1 and CheA4) mix to form the base plate for each of these arrays (19) (Fig. 1a). Both chemoreceptor arrays are still observed by cryo-electron microscopy conducted on multiple *ΔcheA1* or *ΔcheA4* cells, but no array is detected in mutants lacking both CheA1 and CheA4 (here, the *ΔcheA1 ΔcheA4* strain). This organization was confirmed by quantitative fluorescence microscopy and pulldown assays and is thought to provide a mechanism for signal integration through both pathways during chemotaxis (19).

In addition to controlling chemotaxis, previous work has shown that mutations in Che1 affect nonchemotaxis responses: clumping and cell length (7, 12). In *A. brasilense*, CheA1 is produced in two isoforms: a membrane-bound isoform with a conserved seven-transmembrane domain of unknown function (TMX) at the N terminus and a soluble isoform. The membrane-bound isoform is responsible for control of cell length by an unknown mechanism, while the soluble isoform functions in chemotaxis and clumping (8, 10). The role of CheA1 in clumping may result from its role in controlling transient changes in swimming speed (20), which produces, together with changes in swimming reversals controlled by CheA4, the chemotaxis response in *A. brasilense* (8). Both the soluble CheA1 isoform and CheA4 possess prototypical domains found in homologous CheA proteins from other chemotactic and motile bacteria (1–4). To gain further insight into how CheA1 and CheA4 affect *A. brasilense* physiology, we characterize mutants lacking CheA1 (while also excluding effects caused by the nonchemotaxis membrane-bound isoform of CheA1) or CheA4 or both CheA1 and CheA4 using proteomics and metabolomics and physiological and behavioral assays. Proteomics is a powerful method used for the identification and quantification of proteins (through identification of derived peptides) expressed within a cell and thus it provides a snapshot of genome expression (21). Metabolomics allow the quantitative evaluation of a large set of metabolites within a cell providing a direct readout of a cell physiological state (22, 23). Both metabolomics and proteomics use mass spectrometry (MS) coupled to different chromatographic separation technique tools to analyze a large number of peptides (proteomics) or metabolites (metabolomics) simultaneously.

Results obtained here using these technologies reveal that CheA1 and CheA4 alter the abundance of nonchemotaxis proteins implicated in various cellular functions and a subset of metabolites that together point to alteration of nitrogen metabolism. Physiological assays confirm the distinct growth and survival of mutants lacking CheA1, CheA4, or both CheA1 and CheA4 in the presence of nitrate as a nitrogen source and under nitrogen fixation conditions. Our data suggest CheA1- and CheA4-dependent changes in nitrogen metabolism depend, at least partially, on their effect on the abundance of the global regulator RpoN. Together, our results identify unprecedented functional and regulatory links between chemotaxis and nitrogen metabolism.

## RESULTS

### Overview of the differentially expressed proteins in the proteome of *ΔcheA1*, *ΔcheA4*, and *ΔcheA1 ΔcheA4* mutants relative to the parental strain.

We performed all experiments using a minimal defined medium for *A. brasilense* that includes preferred carbon (malate) and nitrogen (ammonium chloride) sources for *A. brasilense* and supports both motility and chemotaxis in this species (24). This medium has been used in all past studies that characterized *A. brasilense* chemotaxis behavior and underlying molecular mechanisms by our group (7, 8, 9, 15, 25). Since the CheA1 histidine kinase is synthesized in two isoforms in our laboratory’s strain of *A. brasilense* Sp7 (10), we first identified the subset of proteins solely dependent on the presence of the soluble chemotaxis CheA1 isoform (*cheA1Δ*TMX). We compared the proteomes of the parental strain Sp7 (expressing an empty vector as a control, pBBR) with that of the *ΔcheA1* strain expressing an empty vector (pBBR), a full-length *cheA1* (pBBRCheA1), or a *cheA1*ΔTMX (pBBRCheA1ΔTMX) (see Table S1 in the supplemental material). We assigned proteins as dependent on the presence of the CheA1 membrane-bound isoform as those uniquely expressed in both the *ΔcheA1*(pBBR) and the *ΔcheA1*(pBBRCheA1) proteomes but not in the proteome of the *ΔcheA1*(pBBRCheA1*Δ*TMX) strain (see Table S1). This subset of proteins was removed from comparison in subsequent analyses. Therefore, the proteins identified as differentially affected in *ΔcheA1* include only proteins whose expression was not affected by TMX.

10.1128/mSystems.01354-20.2 TABLE S1 Raw proteome data for *A. brasilense* Sp7(pBBR1) and *ΔcheA1*(pBBR1CheA1ΔTMX) strains. Download Table S1, XLSX file, 2.6 MB. Copyright © 2021 Ganusova et al. 2021 Ganusova et al. https://creativecommons.org/licenses/by/4.0/ This content is distributed under the terms of the Creative Commons Attribution 4.0 International license.

**FIG 2:**
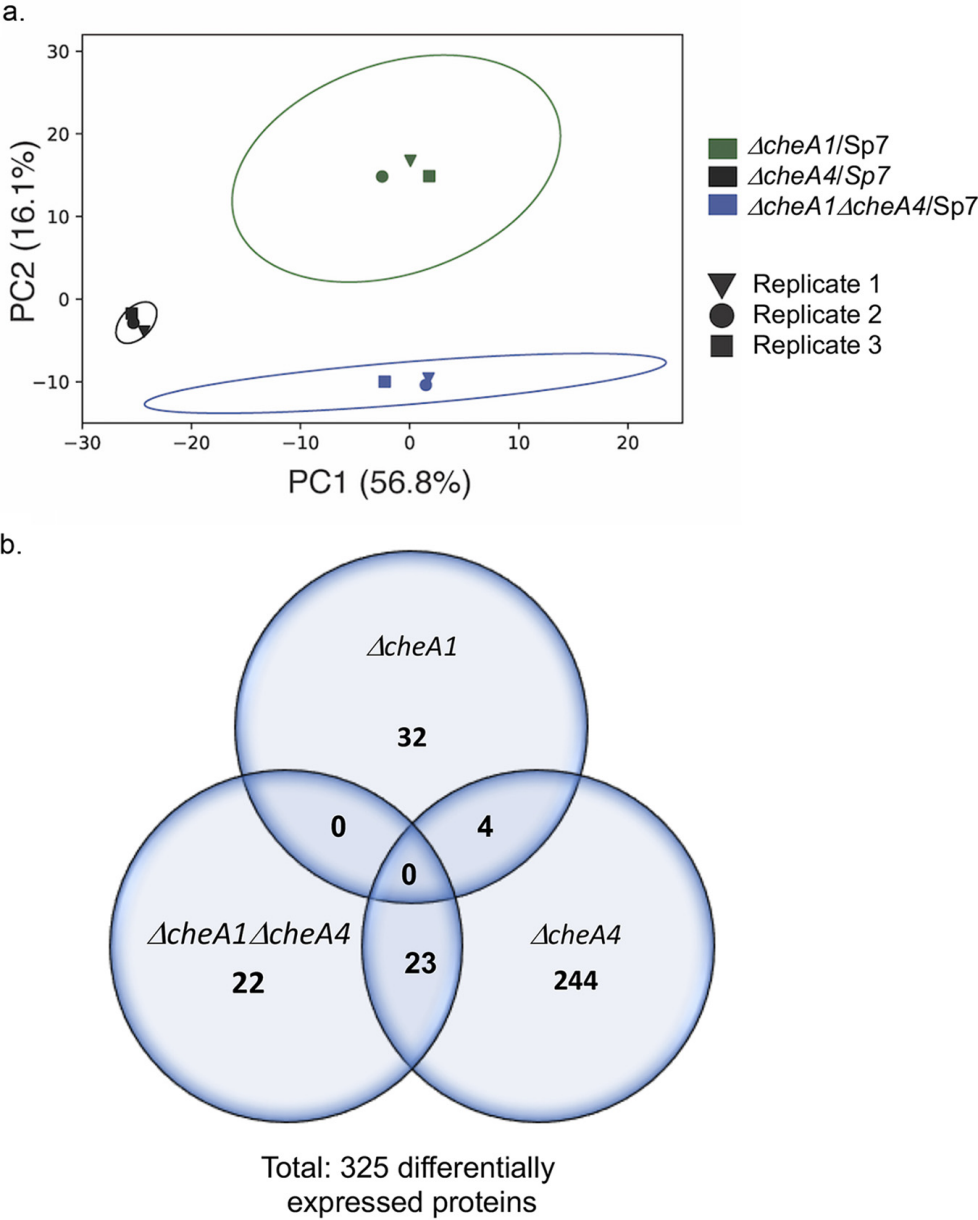

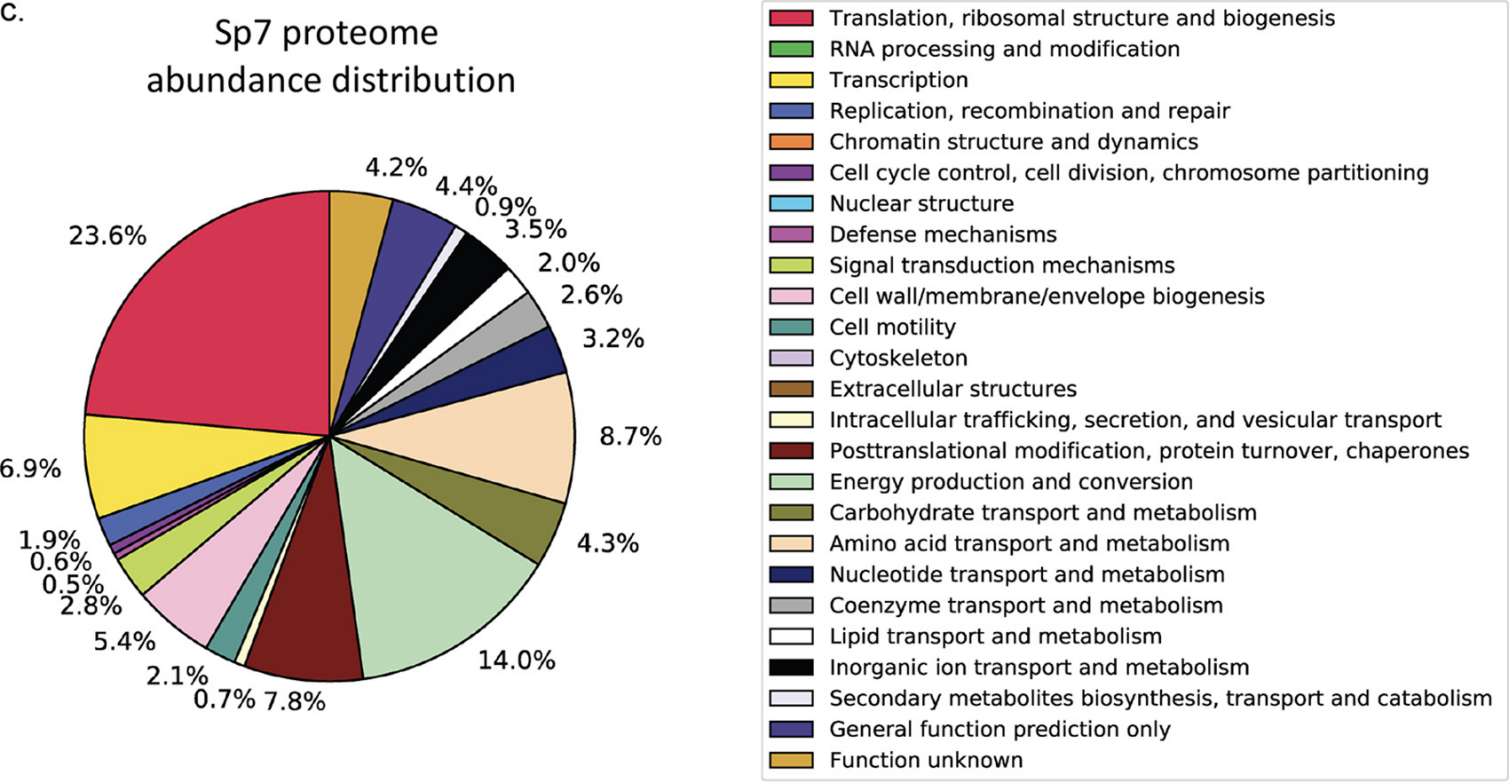
Biplots from 2D PCA of log_2_-fold changes in differentially expressed proteins from *ΔcheA1* versus wild-type (WT; Sp7) (green), *ΔcheA4* versus WT (Sp7) (black), and *ΔcheA1 ΔcheA4* versus WT (Sp7) (blue) strains. (a) Each plot was built based on three biological replicates per strain. Each scatter point, in respective colors, represents the score values of the principal components, and 95% confidence intervals of the score values are modeled as ellipses. (b) Venn diagram representing numbers of the differentially expressed proteins detected through analyses of proteomics data of *ΔcheA1*, *ΔcheA4*, and *ΔcheA1 ΔcheA4* mutants. (c) Functional classification and representation of *A. brasilense* Sp7 proteome in the present study.

A principal-component analysis (PCA) revealed that the proteomes of all strains compared to wild type were distinct and did not overlap (Fig. 2a). In total, 325 proteins were differentially expressed in each mutant compared to the wild type, out of a total of 2,328 proteins predicted to be encoded in the *A. brasilense* genome. Of these 325 proteins, 36, 271, and 45 protein sequences belonging to differentially expressed proteins were identified in the *ΔcheA1*, *ΔcheA4*, and *ΔcheA1 ΔcheA4* mutant proteomes, respectively (Fig. 2b). No differentially expressed protein was common to all three mutant strains or between the proteomes of the *ΔcheA1* and the *ΔcheA1 ΔcheA4* mutants, whereas only 4 proteins were common to both the *ΔcheA1* and *ΔcheA4* mutant proteomes and 23 proteins were common to the *ΔcheA4* and *ΔcheA1 ΔcheA4* mutant proteomes. This distribution suggests that the lack of CheA4 has the greatest effect on proteome composition. The limited or lack of shared differentially expressed proteins between the proteomes of the single mutants with that of the double mutant further suggests distinct roles for CheA1 and CheA4 in mediating these proteome changes since they are not recapitulated in the double mutant strain. Another nonexclusive explanation is that these effects are mediated through intact chemotaxis signaling arrays, which are present in a *ΔcheA1* mutant and a *ΔcheA4* mutant but are absent in a *ΔcheA1 ΔcheA4* mutant (13). The MS-based identification of proteins used in this study depends on the generation of peptides through trypsin digestion prior to separation and has inherent technical limitations. Some proteins may be present in the analyzed samples but may not be detected due to limited or partial protein digestion, peptide signals that fall below detection limits, poor retainment or separation by liquid chromatography or unfavorable electrospray ionization. In addition, various protein modifications can also cause inefficient protein detection by MS (21, 26). Therefore, lack of detection of a protein does not equate to its absence in the samples. Proteins compared here include those detected in all proteomes.

The COG (“clusters of orthologous groups”) term distribution of proteins expressed in the wild-type Sp7 under the conditions of our experiments indicates a significant increase in proteins from translation, ribosomal structure, and biogenesis, as well as energy production and conversion, and a reduction in signal transduction mechanisms compared to the COG term distribution predicted from the genome sequence (Fig. 2c). This result is consistent with active growth of the Sp7 strain under conditions used for proteomics analysis.

### Detection of chemotaxis proteins in the *A. brasilense* wild-type (Sp7) proteome.

**FIG 3:**
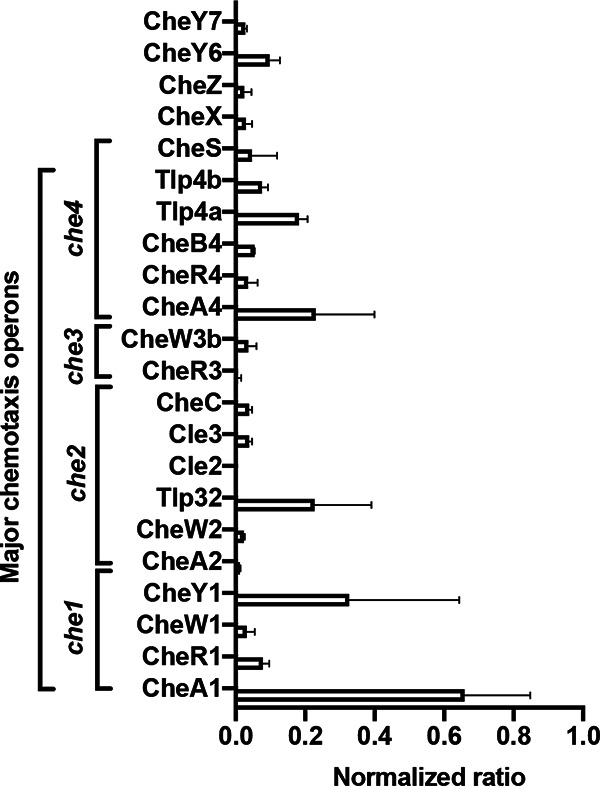
Proteomic analysis of the chemotaxis proteins in the *A. brasilense* Sp7 strain. The protein abundance of all detected chemotaxis proteins was normalized to the protein abundance of CheA4. The normalized abundance of the proteins expressed from the chemotaxis genes and operons *che1*, *che2*, *che3*, and *che4* is depicted.

The proteomics data provided an opportunity to identify chemotaxis and chemosensory signaling protein(s) expressed under the experimental conditions used in the study. Of 90 predicted chemotaxis proteins annotated in the *A. brasilense* Sp7 genome (27), we identified 42 chemotaxis proteins: 18 of these were encoded in the chemosensory pathways (Che1, Che2, Che3, and Che4), including the two chemotaxis pathways (Che1 and Che4) known to regulate the motility pattern and two response regulators (CheY6 and CheY7) encoded elsewhere in the *A. brasilense* genome. We next evaluated the stoichiometry of chemotaxis proteins by normalizing the abundance of these proteins to the abundance of GlyA. We selected GlyA because the corresponding gene, *glyA* serves as a validated housekeeping gene in quantitative PCR studies in *A. brasilense* (28), and it is detected in all the proteomes compared here (Fig. 3). This analysis detected 4 of 5 proteins encoded in the *che1* operon (CheA1, CheW1, CheR1, and CheY1), 6 of 9 proteins predicted in the *che4* operon (CheA4, CheB4, CheR4, CheS, Tlp4a, and Tlp4b), 6 of 10 proteins encoded in the *che2* operon (CheA2, CheW2, Cle2, Cle3, CheC, and Tlp32), and 2 of 8 proteins in the *che3* operon (CheW3b and CheR3) (Fig. 1b). Under these conditions, CheA1 is the most abundant chemotaxis protein, followed by CheY1, CheA4, CheY6, and CheR1, with all others demonstrating relatively low abundances (Fig. 3).

Of the 51 predicted chemoreceptors in the *A. brasilense* genome, we detected 26 such proteins in the wild-type proteome. Most detected chemoreceptors (20 of 26) are predicted to belong to the 38H length class, which is the most abundant class of chemoreceptors predicted to be encoded in the *A. brasilense* Sp7 genome (13). The analysis of chemotaxis proteins by proteomics thus indicates that chemotaxis proteins from all four Che systems and diverse chemoreceptor proteins are expressed under the conditions of our study, albeit at various abundances.

### Cluster analysis of differentially expressed proteins in the wild type compared to mutant proteomes.

**FIG 4:**
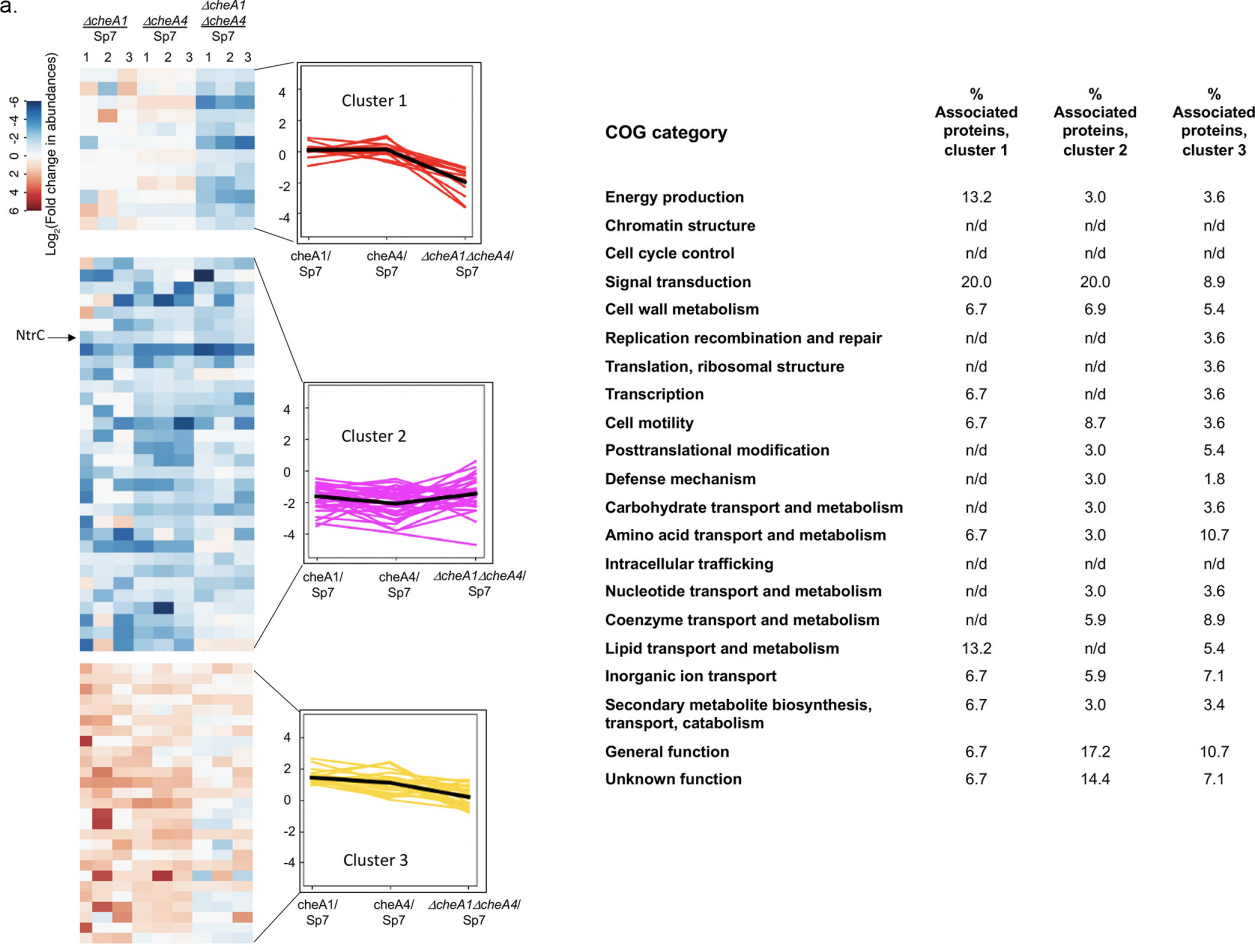

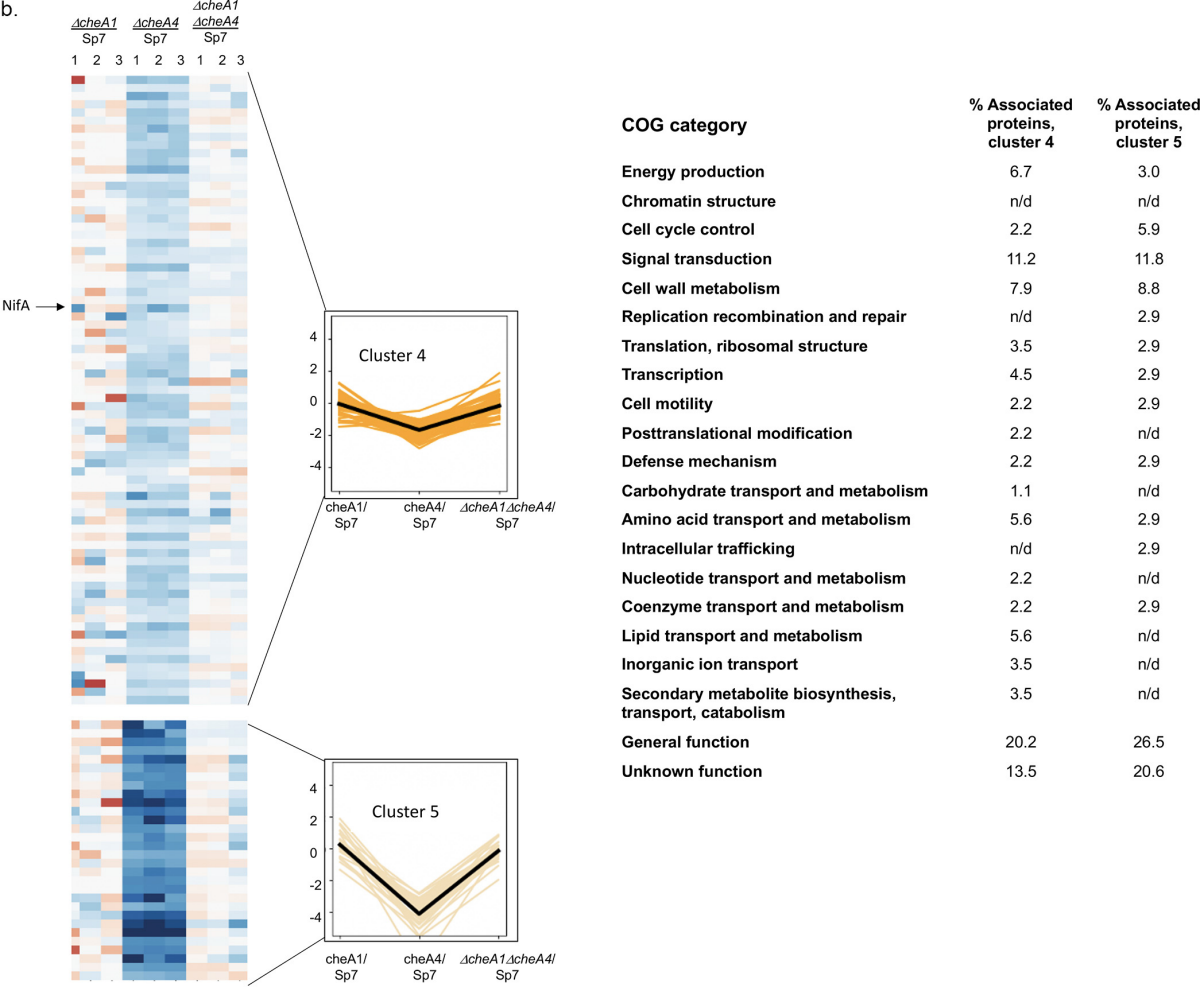

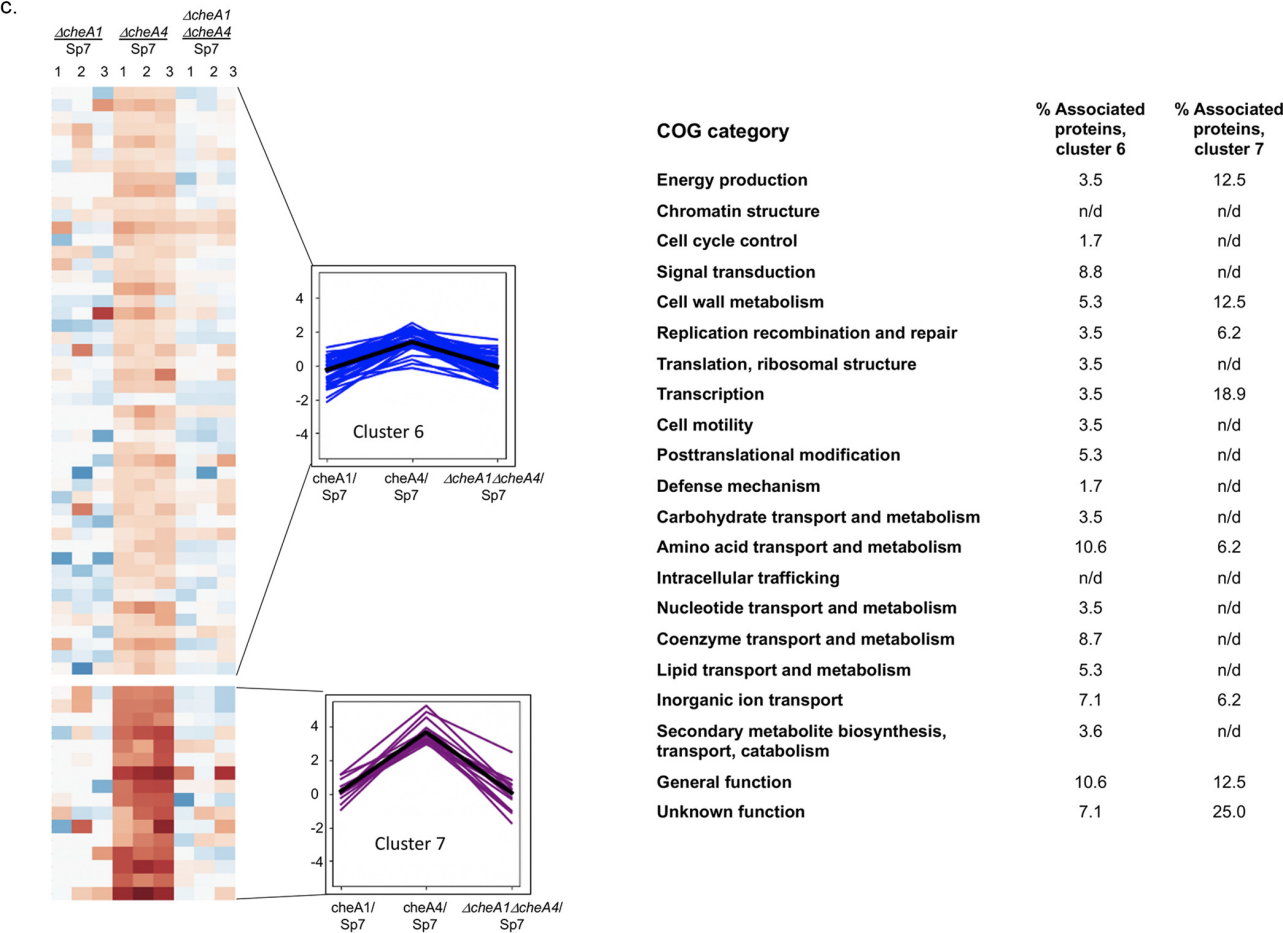

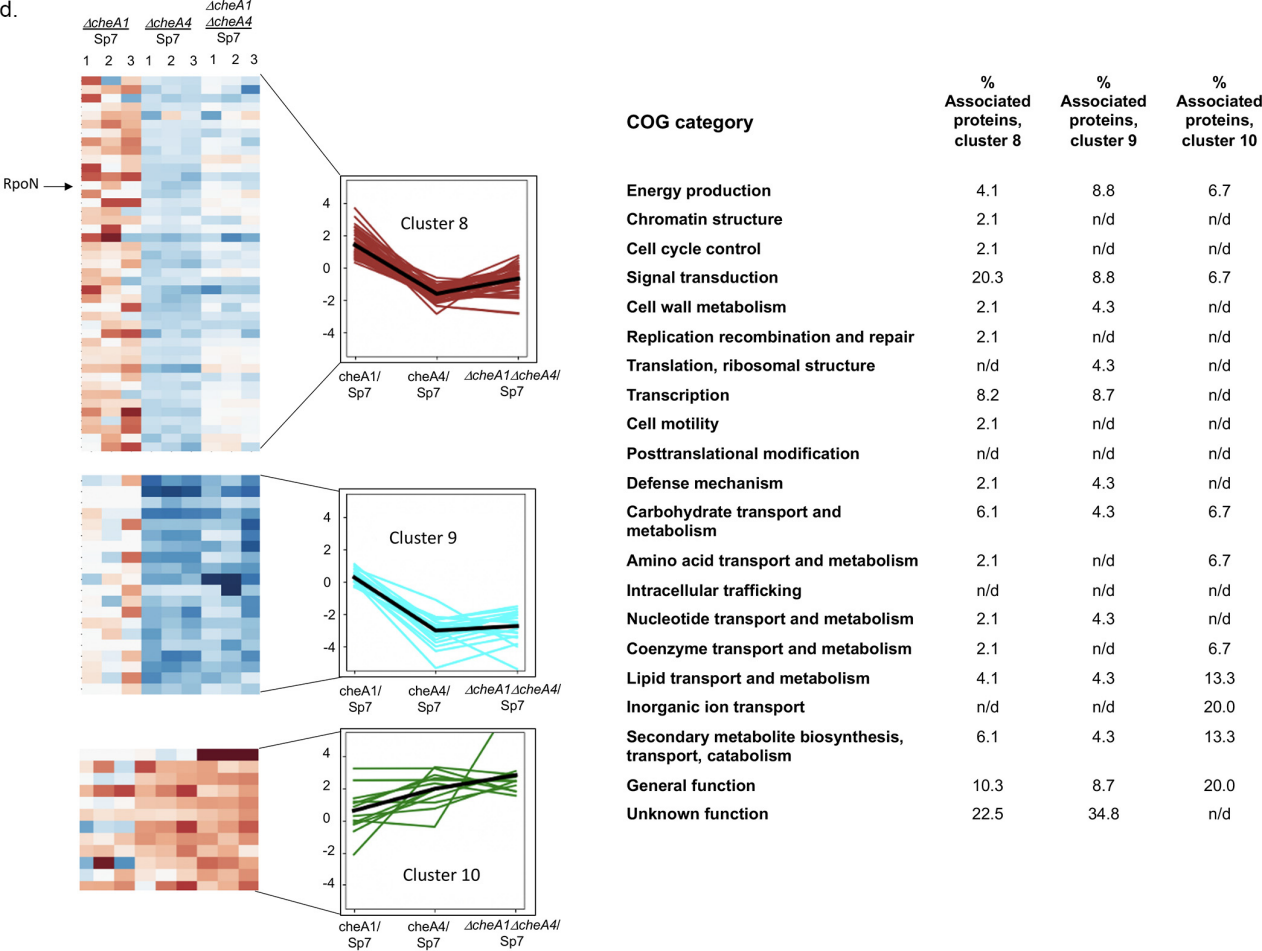
Heatmap representing the hierarchical clustering analysis of differentially expressed proteins in the proteomes of *ΔcheA1* versus wild-type (WT; Sp7), *ΔcheA4* versus WT, and *ΔcheA1 ΔcheA4* versus WT strains, respectively. Protein clusters were built according to the levels of protein expression affected by a single *cheA1* or *cheA4* deletion or a double *cheA1 cheA4* deletion using an averaged log_2_-fold change of expression, as detailed in Materials and Methods. The COG term distribution is shown next to each cluster. The average fold changes for all comparisons for each cluster are also represented as line plots to visualize expression patterns: clusters 1, 2, and 3 (a); clusters 4 and 5 (b); clusters 6 and 7 (c); and clusters 8, 9, and 10 (d). n/d, not detected.

The proteome data sets comparing the wild type to each of the mutants were used to generate a hierarchical cluster analysis in order to identify groups of proteins with similar patterns of abundance variability changes that could suggest they are functionally related (Fig. 4). Overall, we identified 10 clusters. Proteins within each of these clusters were also assigned to COG-based functional categories. The clusters are discussed below based on the type of patterns of abundance changes they represent across the strains.

Cluster 1 represents proteins with decreased expression only in the *ΔcheA1 ΔcheA4* mutant background relative to the wild type (Fig. 4a). This cluster includes proteins related to signal transduction mechanisms (20.0%), followed by lipid transport and metabolism and energy production and conservation (both 13.3%). Among other affected proteins (6.7%) were proteins with function in cell motility, cell wall/membrane/envelope biogenesis, transcription, replication and ribosome structure, amino acid transport and metabolism, secondary metabolite, and inorganic transport and metabolism, as well as proteins of unknown function. Only two chemotaxis proteins were in lower abundance, a putative chemotaxis-specific phosphatase (A0A0POEW78), which is not genetically linked to either *che1* or *che4*, and a transmembrane chemoreceptor (A0A0P0ENJ2), the function of which is not known (see Tables S1 and S2). Among others affected (6.7%) were proteins with function in cell motility, cell wall/membrane/envelope biogenesis, transcription, replication and ribosome structure, amino acid transport and metabolism, secondary metabolite, and inorganic transport and metabolism, as well as proteins of unknown function. These changes indicate that lack of both CheA1 and CheA4, which together control chemotaxis in *A. brasilense*, impacts cell physiology and signaling beyond chemotaxis.

10.1128/mSystems.01354-20.3 TABLE S2 Raw proteome data for *A. brasilense* Sp7, *ΔcheA4*, and *ΔcheA1 ΔcheA4* strains. Download Table S2, XLSX file, 0.1 MB. Copyright © 2021 Ganusova et al. 2021 Ganusova et al. https://creativecommons.org/licenses/by/4.0/ This content is distributed under the terms of the Creative Commons Attribution 4.0 International license.

Cluster 2 represents proteins which abundance is decreased in the three chemotaxis mutants relative to the proteome of the wild type (Fig. 4a). These proteins belong to clusters of orthologous groups (COGs) related to signal transduction mechanisms, cell motility, as well as proteins of general and unknown functions. Among the proteins with lower abundance in these strains are the global enhancing binding protein NtrC (A0A060DLR1) (see Table S1). A lower abundance of NtrC would be expected to alter nitrogen metabolism (25) and suggests that mutations in *cheA1* and/or *cheA4* ultimately affects a major regulator of nitrogen metabolism in this species.

Cluster 3 represents proteins with moderate increased abundance in the *ΔcheA1* and *ΔcheA4* proteomes and remaining unchanged in the double mutant proteome relative to the wild-type proteome (Fig. 4a). This cluster includes proteins involved in amino acid transport and metabolism, signal transduction, and general physiological function and further suggests roles for CheA1 and CheA4 in regulating signaling and nitrogen (amino acid) metabolism.

Clusters 4 and 5 represent proteins with decreased abundance in the *ΔcheA4* mutant (Fig. 4b). Both clusters are mostly represented by proteins with general and unknown functions (20.2 and 26.5%, respectively), followed by signal transduction proteins (11.2 and 11.8%), including chemoreceptors and chemotaxis proteins (A0A0P0EK73, A0A0P0ESU9, A0A0N7I7V7, UPI00070618F0, and A0A0P0EDE4), as well as proteins involved in cell wall/membrane/envelope biogenesis. The nitrogen fixation-specific transcriptional regulator NifA (A0A0P0F6K0) is also present in this cluster despite its expected low expression level under the growth conditions used for proteomics since *nifA* transcription is strongly repressed by ammonium and oxygen in *A. brasilense* (29). The findings suggest basal level of NifA under conditions of growth in the presence of the preferred nitrogen source (ammonium chloride) and oxygen is further reduced in a Δ*cheA4* mutant background. CheA4 may thus affect the abundance and/or stability of NifA under these conditions.

Cluster 6 and 7 represent proteins with moderate to severe elevated protein abundance in the *ΔcheA4* mutant compared to the wild type proteome (Fig. 4c). Proteins with elevated abundance in these clusters span multiple COGs related to signal transduction, transcription, carbon, amino acid and inorganic ions transport and metabolism. In addition to changes in abundance of several chemotaxis proteins in cluster 6 (AerC [A0A0P0E918], A0A0P0F5Y8, UPI000550DB68, UPI00039FB3F7, and A0A0P0F123), affected proteins include uncharacterized transcriptional regulators and several proteins related to ABC transport systems. The soluble chemoreceptor AerC is involved in redox sensing during chemotaxis in gradients of air in *A. brasilense* and has a major role under nitrogen fixation conditions (15). A glutamine synthase (A0A0P0ESU5), distinct from GlnA characterized in *A. brasilense* (30), is also more abundant in the *ΔcheA4* strain proteome. This protein has not been characterized, but our observation of the changes in nitrogen metabolism in the *ΔcheA4* strain suggests that it may play an important role in regulating nitrogen metabolism in *A. brasilense*. Clusters 4, 5, 6, and 7 thus represent proteins which are specifically affected by lack of CheA4. The diversity of the COGs represented in these clusters highlight the pleiotropic impact that CheA4 alone has on cell physiology.

Cluster 8 represents proteins which abundance increases in the *ΔcheA1* strain proteome, decreases in the *ΔcheA4* strain and is only slight changed, if at all, in the *ΔcheA1 ΔcheA4* strain, relative to the wild-type proteome (Fig. 4d). This cluster represents mostly changes in signal transduction (20.3%), proteins of unknown function (22.5%) and of general function (10.3%), as well as transcription and carbohydrate transport and metabolism. In addition to ABC transporters that have not yet been characterized, several transcriptional regulators are affected. The RpoN protein (Q59085) that encodes the alternative RNA polymerase σ^54^ was among the proteins with differential abundance in this cluster (see Tables S1 and S2). In *A. brasilense*, RpoN was previously shown to regulate nitrogen fixation, ammonium uptake, nitrate assimilation, and motility (31). The opposite effects of mutations in *cheA1* or *cheA4* on the abundance of RpoN that are no longer detected in the double mutant further suggest a functional link between chemotaxis and RpoN.

Cluster 9 represents proteins with decreased abundance in the *ΔcheA4* and the *ΔcheA1 ΔcheA4* mutants, indicating that changes are mostly due to absence of CheA4. The majority of proteins detected in this cluster have unknown functions, followed by signal transduction proteins (with no changes in the expression of chemotaxis-related proteins), transcription, energy production and conservation, and proteins with general function (Fig. 4d).

Finally, cluster 10 comprises proteins with variable abundance changes but trending toward an elevated abundance in the order of *ΔcheA1*, *ΔcheA4*, and *ΔcheA1 ΔcheA4* proteomes relative to the wild type. Proteins in this cluster include those related to general function, lipid transport and metabolism, inorganic ion transport and metabolism, and secondary metabolite biosynthesis, transport, and catabolism (Fig. 4d). Together, these changes suggest that perturbation of chemotaxis signaling leads to pleiotropic effects on metabolism.

### Metabolomics reveal distinct metabolites in the mutants relative to the wild type.

**FIG 5:**
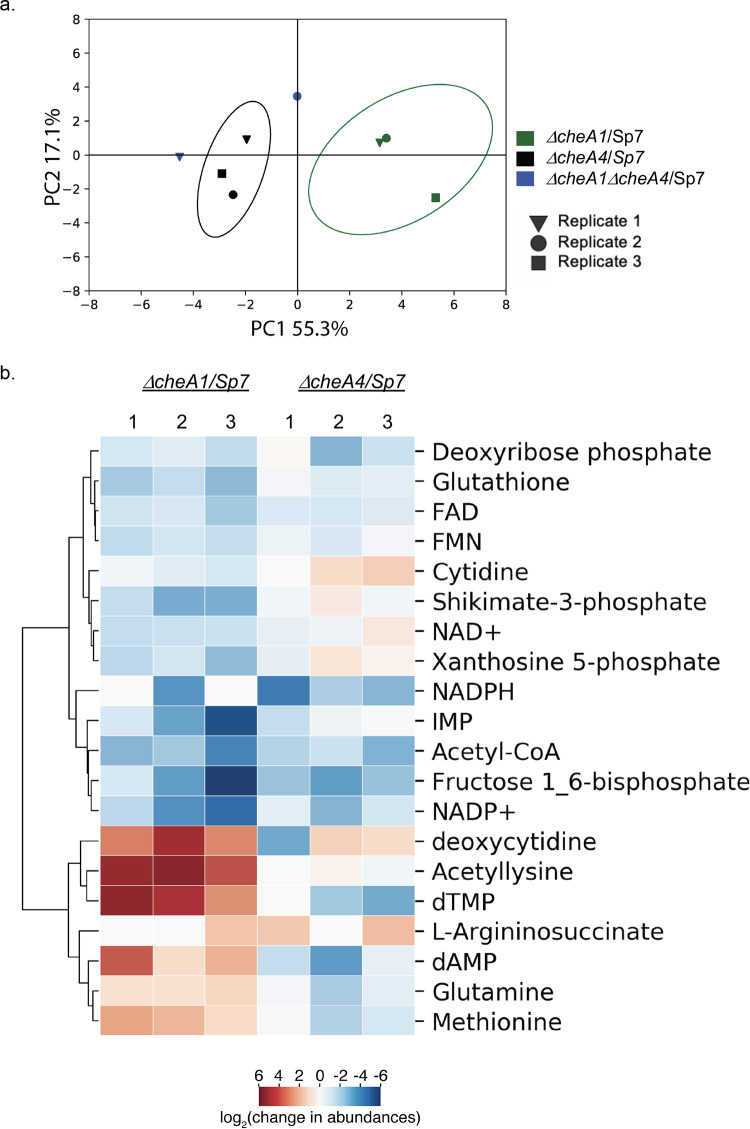
Comparison of the *A. brasilense ΔcheA1*, *ΔcheA4*, and *ΔcheA1 ΔcheA4* mutants using whole-cells metabolomics. (a) Biplots from 2D PCA of log_2_-fold changes indicate significantly different metabolite abundances from *ΔcheA1* versus wild-type (WT; Sp7) (green), *ΔcheA4* versus WT (Sp7) (black), and *ΔcheA1 ΔcheA4* versus WT (Sp7) (blue) strains. Metabolite identification and statistical representation are shown. (b) Cluster heat map visualization of the significantly different metabolite abundances in Δ*cheA1* and Δ*cheA4* strain replicates compared to the wild type (Sp7).

We were able to detect a total of 106 metabolites in Sp7 and mutant chemotaxis strains that were matched to known compounds in libraries (see Table S3). A principal-component analysis (PCA) revealed that the metabolome of *ΔcheA1* and *ΔcheA4* strains were distinct from one another and to that of the wild-type strain, suggesting different metabolic status for these strains (Fig. 5a). The *ΔcheA1 ΔcheA4* metabolome sample replicates did not group together or with either single mutant and they appeared highly variable (Fig. 5a). One possibility may be that only two replicates were included for the *ΔcheA1 ΔcheA4* strain, which could have led to an inability to capture any pattern using a statistical method such as PCA. Only 20 metabolites had significant abundance changes in the single chemotaxis mutants in comparison with the wild-type strain Sp7 (Fig. 5b). A cluster heatmap analysis revealed two clusters of metabolites with opposite abundance in the *ΔcheA1* in the Δ*cheA4* mutant strain relative to the wild type. The first cluster includes metabolites with lower abundance in both *ΔcheA1* and *ΔcheA4* mutants, with a few exceptions (Fig. 5b). This cluster is represented by metabolites detected in gluconeogenesis and glycolysis, fructose-1-6-bisphosphate and acetyl coenzyme A, as well as glutathione which plays a role in maintaining intracellular redox, shikimate-3-phosphate (reduced in all three *ΔcheA1* sample replicates but not all of the *ΔcheA4* mutant sample replicates), a precursor for aromatic amino acids, the pyrimidine cytidine (reduced in all three *ΔcheA1* sample replicates but not in all of the *ΔcheA4* mutant sample replicates), and coenzymes such as flavin mononucleotide (FMN) and flavin adenine dinucleotide (FAD), as well as reducing forms of NAD such NAD^+^ (reduced in all three *ΔcheA1* sample replicates but not all of the *ΔcheA4* mutant sample replicates), NADP^+^, and NADPH. The same pattern of abundance was detected for metabolites implicated in purine and pyrimidine metabolism, xanthosine-5-phosphate (reduced in all three *ΔcheA1* sample replicates but not all of the *ΔcheA4* mutant sample replicates), IMP, and deoxyribose phosphate. Thus, absence of both major chemotaxis proteins CheA1 and CheA4 affects metabolites that include cofactors and major metabolic intermediates in central carbon metabolism, as well as purine and pyrimidine amine bases that serve in DNA and RNA metabolism.

10.1128/mSystems.01354-20.4 TABLE S3 Raw metabolomics data. Download Table S3, XLSX file, 0.03 MB. Copyright © 2021 Ganusova et al. 2021 Ganusova et al. https://creativecommons.org/licenses/by/4.0/ This content is distributed under the terms of the Creative Commons Attribution 4.0 International license.

The second cluster included metabolites with high abundance in the *ΔcheA1* and low abundance in the *ΔcheA4* mutant strains relative to the wild type. This cluster included purines and pyrimidines such as dAMP, deoxycytidine (increased in all three Δ*cheA1* sample replicates but not all of the Δ*cheA4* mutant sample replicates) and dTMP, l-arginosuccinate (unchanged or increased in all three Δ*cheA1* sample replicates and in the *ΔcheA4* mutant sample replicates), which functions to replenish the tricarboxylic acid (TCA) cycle, as well as the amino acids acetyllysine, glutamate, and methionine. These observations corroborate differences in the proteomes of the *ΔcheA1* and *ΔcheA4* strains relative to each other and to the wild type, and they suggest distinct physiologies of these strains. The results also identify a subset of changes that are affected in opposite manner in these strains compared to the wild type. Together, these findings indicate a major role for CheA1 and CheA4 in *A. brasilense* physiology that is beyond their established roles in chemotaxis.

### Mutants lacking CheA1 and CheA4 have altered cell physiology and nitrogen metabolism.

To validate some of the hypotheses generated through the proteomics and metabolomics analyses, we next characterized the physiology of the chemotaxis mutants. We have previously showed that *A. brasilense ΔcheA1* or *ΔcheA4* mutants have minor or severe impairments, respectively, in chemotaxis, with these defects complemented by expression of parental genes from broad-host-range vectors (as shown in Fig. S1 in the supplemental material), corroborating similar results in previous studies from our group (7, 9, 10).

10.1128/mSystems.01354-20.1 FIG S1 Chemotaxis behaviors of Sp7(pBBR1), *ΔcheA1*(pBBR1), and *ΔcheA1*(pBBR1CheA1*Δ*TMX) (a) and Sp7(pRK415), *ΔcheA4*(pRK415), and *ΔcheA4*(pRK415CheA4) (b) mutants of *A. brasilense* and complementation in the soft agar plate assay containing malate (10 mM) and ammonium chloride (18.7 mM) as the carbon and nitrogen sources, respectively. The strains tested are indicated at the top of each plate. Swim zone diameters were measured after 5 days of incubation at 28°C. Asterisks indicate statistically significantly different values compared to the value for the wild-type strain (Student *t* test; *, *P* < 0.01, **, *P* < 0.005, ***, *P* < 0.001). In the left panel, “***” represents statistical significance between *ΔcheA1*(pBBR1) and Sp7(pBBR1) (a) and “***” represents statistical significance between *ΔcheA1*(pBBR1CheA1ΔTMX) and *ΔcheA1*(pBBR1) (b). In the right panel, “*” represents statistical significance between *ΔcheA4*(pRK415) and Sp7(pRK415) (a) and “****” represents statistical significance between *ΔcheA4*(pRK415CheA4) and *ΔcheA4*(pRK415) (b). Download FIG S1, PDF file, 0.06 MB. Copyright © 2021 Ganusova et al. 2021 Ganusova et al. https://creativecommons.org/licenses/by/4.0/ This content is distributed under the terms of the Creative Commons Attribution 4.0 International license.

Next, we compared *ΔcheA1*, *ΔcheA4*, and *ΔcheA1 ΔcheA4* mutants for a range of physiological features. First, we determined the growth rates and lag times for the different mutants and their complemented strain derivatives using the same conditions as those used for proteomics (minimal medium, with malate as a carbon source and ammonium chloride as a nitrogen source) (Table 1). We found that the growth rate of all the mutants did not differ significantly from that of the wild type. The major difference was in the greater lag time of the *ΔcheA1* and the *ΔcheA1 ΔcheA4* mutant strains. The extended lag phase of the *ΔcheA1* strain relative to the wild type could be complemented, albeit partially, by expression of the CheA1ΔTMX variant from a plasmid. We have previously observed similar partial complementation when expressing genes from plasmids (7, 9, 10, 12). These results suggest different metabolic capacities, especially in the *ΔcheA1* and *ΔcheA1 ΔcheA4* mutants, relative to the wild-type strain Sp7.

**TABLE 1:** Effect of *ΔcheA1*, *ΔcheA4*, and *ΔcheA1 ΔcheA4* deletions on the growth lag phase of *A. brasilense* in MMAB medium with ammonium chloride as a nitrogen source

Strain	Mean lag time (min) ± SEM
Sp7	156.7 ± 19.7
*ΔcheA1*	480 ± 17.9
*ΔcheA4*	163.3 ± 32
*ΔcheA1 ΔcheA4*	333.3 ± 89.1
Sp7(pBBR)	273.3 ± 8.4
*ΔcheA1*(pBBR)	335.0 ± 25.3
*ΔcheA1*(pBBRCheA1ΔTMX)	317.1 ± 36.4
Sp7(pRK415)	304.4 ± 17.6
*ΔcheA4*(pRK415)	312.0 ± 19.6
*ΔcheA4*(pRK41CheA4)	303.3 ± 3.3

Because our proteomes data suggested changes in nitrogen metabolism (e.g., different abundances of NifA, NtrC, and RpoN in different mutant strains), we next compared the mutants for growth with different nitrogen sources. First, we compared the growth of the wild type and mutants on media containing nitrate (Table 2). We found that the *ΔcheA1* strain grew faster in the presence of potassium nitrate, while the *ΔcheA4* strain grew slower and the double mutant had an intermediate phenotype. Growth defects of the *ΔcheA1* and Δ*cheA4* mutants under these conditions were complemented by introducing plasmids carrying the full-length of *cheA1*ΔTMX (pBBR1CheA1 ΔTMX) and *cheA4* (pRK415CheA4) (Table 2).

**TABLE 2:** Effect of *ΔcheA1*, *ΔcheA4*, and *ΔcheA1 ΔcheA4* deletions on the growth rate of *A. brasilense* in MMAB medium with potassium nitrate as a nitrogen source

Strain	Mean growth rate (h^−1^) ± SEM
Sp7	0.115 ± 0.003
*ΔcheA1*	0.172 ± 0.006
*ΔcheA4*	0.062 ± 0.002
*ΔcheA1 ΔcheA4*	0.058 ± 0.001
Sp7(pBBR)	0.053 ± 0.001
*ΔcheA1*(pBBR)	0.087 ± 0.017
*ΔcheA1*(pBBRCheA1ΔTMX)	0.045 ± 0.019
Sp7(pRK415)	0.086 ± 0.004
*ΔcheA4*(pRK415)	0.039 ± 0.007
*ΔcheA4*(pRK415CheA4)	0.119 ± 0.070

**FIG 6:**
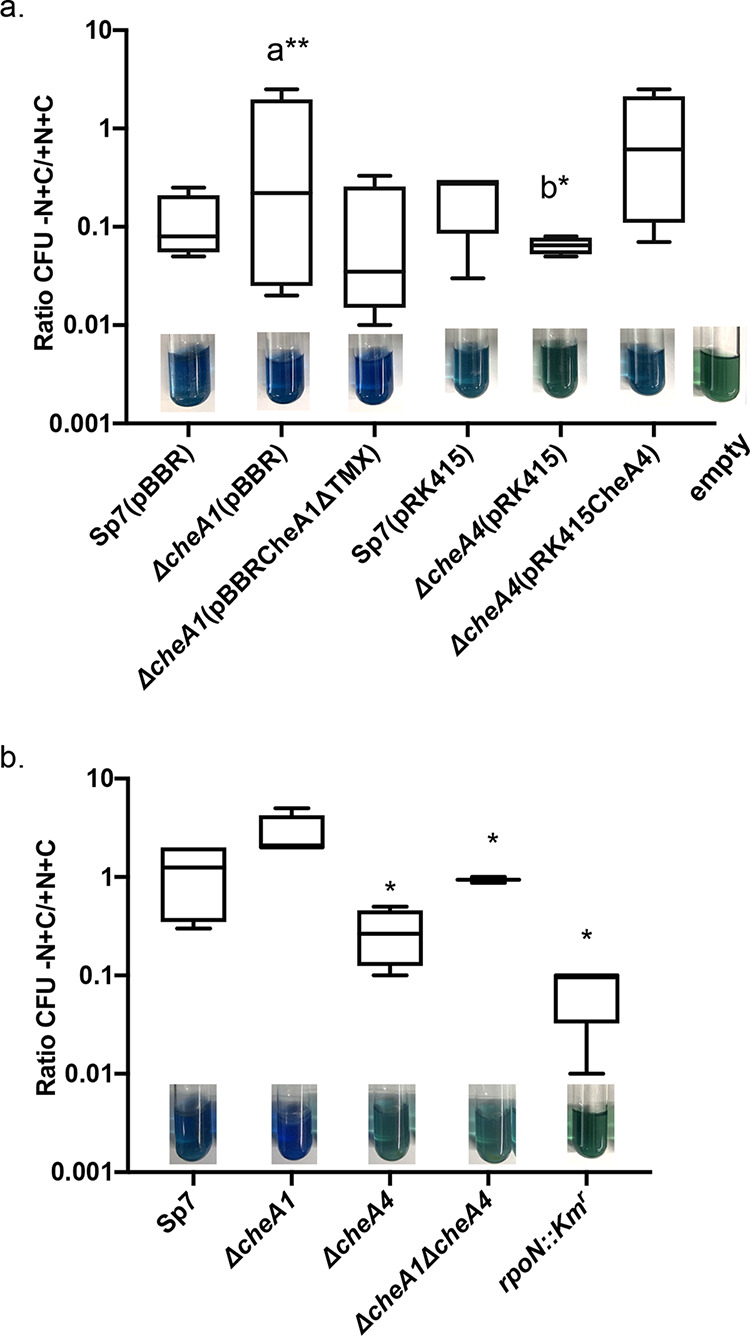
Role of *A. brasilense* CheA1 and CheA4 in nitrogen fixation. (a) Functional complementation of the *ΔcheA1* and *ΔcheA4* mutants under nitrogen fixation conditions with parental genes expressed from broad-host-range plasmids is also shown with strains carrying empty vectors as references. (b) Survival of noncomplemented *ΔcheA1*, *ΔcheA4*, and *ΔcheA1 ΔcheA4* strains under nitrogen fixation conditions versus conditions with available fixed nitrogen source (ammonium chloride). The *rpoN*::Km^r^ strain was used as a control. The values shown are average ratios of CFU recovered from the MMAB without a nitrogen source versus CFU recovered from MMAB with ammonium chloride. Insets show changes in the pH indicator (bromothymol blue) coloration in semiliquid media inoculated with strains. Asterisks indicate statistically significant differences compared to the value for the wild-type strain (Student *t* test; *, *P* < 0.01; **, *P* < 0.05). a**, statistical significance between *ΔcheA1*(pBBR1) and Sp7(pBBR1); b*, statistical significance between *ΔcheA4*(pRK415) and Sp7(pRK415).

We next compared growth under nitrogen fixation conditions, i.e., low aeration to limit oxygen availability and lack of an organic nitrogen source. Chemotaxis in air gradients is essential for *A. brasilense* ability to fix nitrogen (15, 24). Because *ΔcheA4* and *ΔcheA1 ΔcheA4* mutants are null for chemotaxis in gradients of air (9), we needed to compare these strains under conditions where chemotaxis would not confound growth. Therefore, we carried out this assay by spotting washed overnight cultures on plates containing either nitrogen-free minimal medium or medium supplemented with ammonium chloride as a reference since the strains do not have differences in their growth rates in the presence of ammonium chloride. We expressed the results relative to the growth of the strains in the presence of ammonium chloride. As a negative control in these experiments, we used a *rpoN*::Km^r^ mutant derivative of *A. brasilense* which does not fix nitrogen and does not grow under nitrogen fixation conditions that was previously constructed and characterized by others (32). We found that both *ΔcheA4* and *ΔcheA1 ΔcheA4* mutants survived less under conditions of nitrogen fixation compared to the wild type and that the *ΔcheA1* mutant survived better than the wild type under these conditions. These defects were fully complemented by expressing parental genes from plasmids (Fig. 6a). Absence of chemotaxis regulator CheA1 or both CheA1 and CheA4 (*ΔcheA1 ΔcheA4* strain) thus impairs *A. brasilense* ability to grow under nitrogen-fixing conditions (Fig. 6b). We also visualized the nitrogen fixation ability of the mutants and their complemented strains, using a nitrogen-free semisolid minimal medium for *A. brasilense* (MMAB) supplemented with bromothymol blue to detect pH changes that result from bacterial metabolism (here, nitrogen fixation), as described previously (33). The results are consistent with the poor nitrogen fixation ability of the *ΔcheA4* and the *ΔcheA1 ΔcheA4* strains (see inset images in Fig. 6).

### Lack of *cheA1* and/or *cheA4* impacts the activity of a subset of promoters for nitrogen fixation metabolism genes.

**FIG 7:**
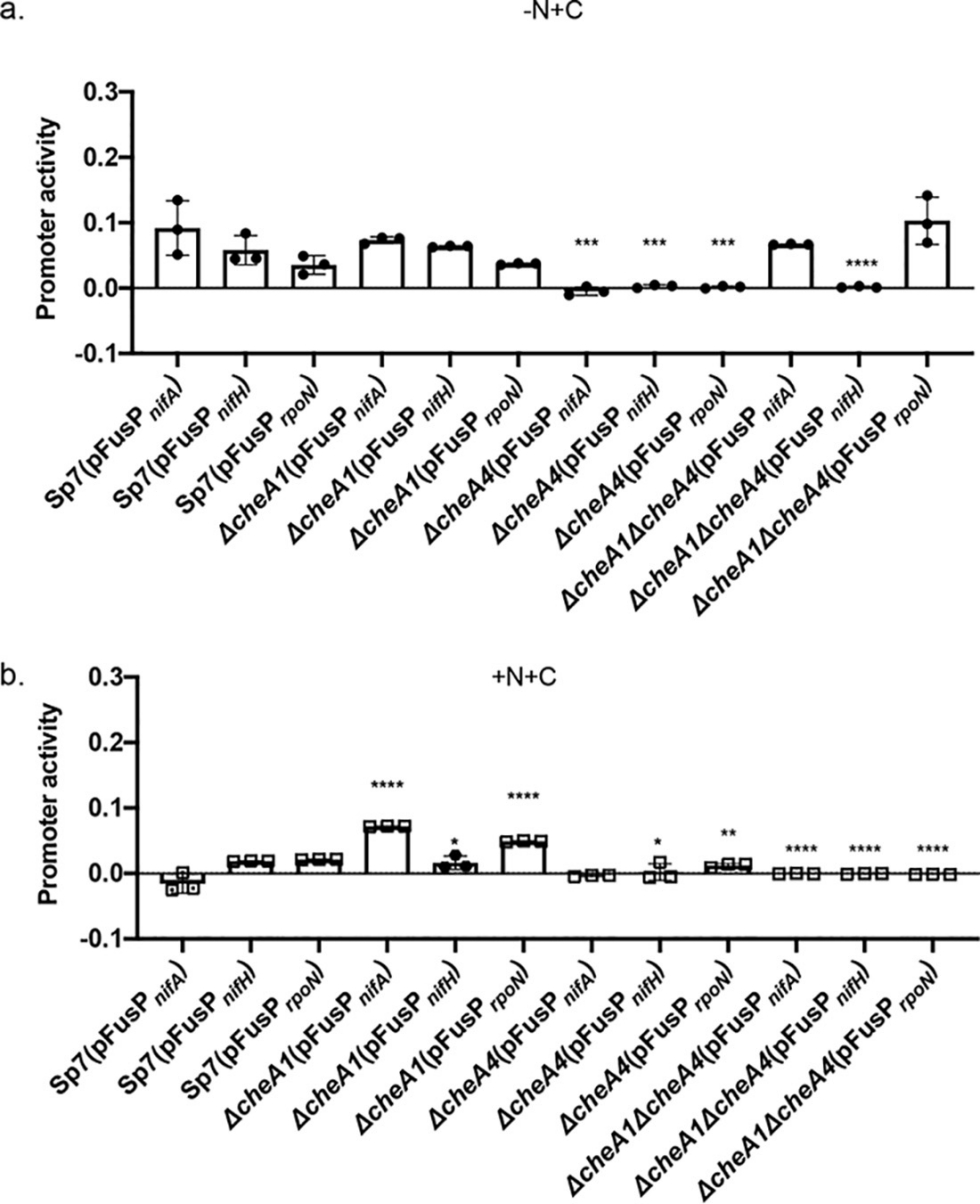
Activity of *nifA*, *nifH*, and *rpoN gusA* promoter fusions in *A. brasilense* wild-type (Sp7) and *ΔcheA1*, *ΔcheA4*, and *ΔcheA1 ΔcheA4* mutant derivatives in MMAB lacking any nitrogen source and under nitrogen fixation conditions (a) or with ammonium chloride as a nitrogen source (b), conditions similar to the proteomics experiments. Values indicating promoter activity were normalized to the values obtained from the cultures containing an empty vector. This calculation was necessary due to variable values obtained with the cells containing an empty vector. Asterisks indicate statistically significant differences compared to the value for the wild-type strain (Student *t* test; *, *P* < 0.01; **, *P* < 0.05; ***, *P* < 0.005; ****, *P* < 0.001).

Free-living nitrogen fixation in *A. brasilense* has been studied, and genes encoding nitrogenase, including the major structural component encoded by *nifH* and several regulators (*rpoN*, *ntrC*, and *nifA* detected here) have been characterized (34–37). In *A. brasilense*, RpoN is a global regulator of nitrogen metabolism, and it regulates the transcription of *nifH* (38), while transcription of *nifA* is maximum in the absence of ammonia and oxygen (29). To confirm the role of CheA1 and CheA4 in affecting nitrogen fixation suggested by our data above, we analyzed promoter activity of the *rpoN*, *nifA*, and *nifH* genes in the wild type and the *ΔcheA1*, *ΔcheA4*, and *ΔcheA1 ΔcheA4* mutants under nitrogen fixation growth conditions (Fig. 7a). *rpoN*, *nifA*, and *nifH* promoter activity in the wild type (Sp7) and the *ΔcheA1* strains was elevated under nitrogen-fixing conditions, as described previously (29, 32, 39) (Fig. 7a). We did not detect a significant elevation of *nifH* or *rpoN* promoter activity in the *ΔcheA1* mutant relative to the wild type that could explain the increased survival and growth of this mutant under conditions of nitrogen fixation and in the presence of nitrate, suggesting that the growth advantage conferred by lack of CheA1 may either be independent of NifA and RpoN or occurs at a posttranscriptional level to modulate the activity of the corresponding proteins. The activity of all three promoters was essentially abolished in the *ΔcheA4* strain, consistent with the reduced ability of this strain to survive under nitrogen fixation conditions. In contrast, the activity of the *nifA* and *rpoN* promoters in the *ΔcheA1 ΔcheA4* strain was comparable to that in the wild type or the *ΔcheA1* mutant, but activity of the *nifH* promoter was absent, consistent with the poor survival of the double mutant under nitrogen-fixing conditions. These results were surprising given that both NifA and RpoN, whose promoters appear active in this strain background, are required for *nifH* transcription (38). The results suggest that the lack of CheA4 alone is responsible for the lack of transcription of *nifH*, even when the promoters for *nifA* and *rpoN* are active. The results further imply that CheA4 may function to increase *nifA* and *rpoN* promoter activity under these conditions.

To further explore these unanticipated effects of chemotaxis mutations on the activity of these promoters, we repeated the experiments on cells grown in the presence of ammonium chloride and with aeration, similar to the experimental conditions used in proteomics (Fig. 7b). As expected from the presence of ammonium chloride and aeration conditions, there was no activity for the *nifH* promoter in all strains under these conditions. However, the activity of the *rpoN* and *nifA* promoters was elevated in the *ΔcheA1* strain and abolished in the *ΔcheA4* and *ΔcheA1 ΔcheA4* mutant strains. The activity of both promoters is thus derepressed in the *ΔcheA1* strain under these conditions compared to the wild-type strain. This pattern of activity is consistent with the elevated abundance of RpoN observed in proteomics for this strain. The increased activity of the *nifA* promoter in the *ΔcheA1* background was in contrast to the overall lower NifA abundance, which also showed variability, in the three proteome sample replicates for this strain relative to that of the wild type (see Tables S1 and S2). The *nifA* promoter activity is thus derepressed in a strain lacking CheA1, but the corresponding protein does not accumulate, suggesting additional effects at the transcriptional and/or posttranscriptional levels that are unique to the Δ*cheA1* mutant background.

## DISCUSSION

In this study, we identified proteomic and metabolic changes caused by knockdowns of the two chemotaxis histidine kinases—CheA1 and CheA4—in *A. brasilense* which together form a functional chemotaxis signaling system in this species. Results from both proteomics and metabolomics clearly demonstrate a role for these proteins in modulating cell physiology beyond chemotaxis. Changes observed in the chemotaxis mutants spanned carbon, amino acid, DNA, RNA, and nitrogen metabolisms, including transport as well as transcription and signal transduction. We confirmed several of these effects of mutating *cheA1* or *cheA4* on metabolism in physiological assays. Chemotaxis and metabolism are tightly coupled in *A. brasilense*, and the strongest chemoeffectors in this species are compounds that affect energy metabolism (24). This coupling has been linked to chemoreceptors that specifically monitor intracellular energy-related parameters such as FAD (13, 15) or by integrating c-di-GMP signaling with chemoreceptor signaling activity (13, 40, 25). The results presented here suggest that the coupling of chemotaxis with metabolism in *A. brasilense* includes broader metabolic changes. Such global metabolic effects and related proteomic changes likely depend on multiple regulators that could act at the level of signaling, as well as transcriptional or posttranscriptional regulation.

We identified NifA, NtrC, and RpoN among regulators of metabolism differentially affected by chemotaxis mutations in our proteomics data set. All three regulators function in the regulation of nitrogen metabolism in *A. brasilense*, specifically the regulation of nitrogen fixation (NifA, RpoN, and NtrC) (31, 32, 29, 38, 41) and nitrate assimilation (NtrC and RpoN) (31). We observed effects of mutations of *cheA1* and/or *cheA4* on the abundance of the corresponding proteins in proteomics experiments conducted in the presence of ammonium chloride and aeration, which both inhibit nitrogen fixation or nitrate assimilation (31). We also found significant differences in the ability of the chemotaxis mutants to grow or survive with nitrate or under nitrogen fixation conditions, indicating that these effects are physiologically relevant. Together, these observations further underscore a functional coupling between chemotaxis and regulators of nitrogen metabolism in *A. brasilense*.

The role of CheA1 and CheA4 in the abundance of RpoN appears to be exerted at the transcriptional level and to depend on nitrogen availability. Compared to the wild-type strain, the activity of the *rpoN* promoter was elevated in the *ΔcheA1* strain, was lower in the *ΔcheA4* strain, and was abolished in a double mutant in the presence of ammonium chloride but not under condition of nitrogen fixation, where *rpoN* promoter activity was comparable in both wild type and the *ΔcheA1* mutant and the double mutant strain and absent in the *ΔcheA4* strain. Similarly, the activity of the *nifA* promoter was also differentially affected by the chemotaxis mutations in a nitrogen-dependent manner. Combined, these results indicate that CheA1 functions to suppress the activity of the *nifA* and *rpoN* promoters in the presence of ammonium chloride or under nitrogen fixation conditions, whereas CheA4 functions to increase the activity of these promoters under these conditions (29, 31, 32). The activity of the *nifA* and *rpoN* promoters in a *ΔcheA1 ΔcheA4* mutant was similar to the *ΔcheA4* strain when ammonium chloride was present and similar to the *ΔcheA1* mutant under nitrogen fixation conditions. It follows that CheA1 and CheA4 roles in modulating the activity of the *nifA* and *rpoN* promoters dominate under nitrogen fixation conditions and in the presence of ammonium chloride, respectively. Relatively little is known about how *nifA* and *rpoN* promoters are regulated in *A. brasilense*, but our results suggest that unidentified regulator(s) whose activity depends on the presence of CheA1 and CheA4 function to regulate the strength of these promoters’ activities. Our data also suggest a role for CheA4 in preventing *nifH* promoter activity even when *nifA* and *rpoN* are active, suggesting yet another level of control of *nifH* promoter activity that remains to be characterized.

The opposite phenotypes of the single mutants in a subset of proteomics, metabolomics and physiological data obtained here, with these effects abolished in a double mutant, are consistent with the opposite effects of CheA1 and CheA4 on nitrogen metabolism regulators above. The global regulator RpoN was among proteins affected in this manner in the proteomes, and it was confirmed through an analysis of *rpoN* promoter activity. Several purine and pyrimidine metabolites, amino acids, and l-arginosuccinate that serves to replenish the TCA cycle needed for the synthesis of a number of precursors of amino acid synthesis were also evidenced in the metabolomes. Similar opposite effects of mutations in *cheA1* and *cheA4* included an altered growth rate in the presence of nitrate and survival and growth under nitrogen fixation conditions. Given the role of RpoN in regulation of nitrate assimilation and nitrogen fixation in *A. brasilense* (31), these findings suggest that CheA1 and CheA4 alter nitrogen metabolism at least in part through modulation of the abundance of RpoN. In other bacterial species, RpoN regulates the expression of genes involved in extracellular polymeric substance (EPS) production and biofilms (42, 43), flocculation (44), cell motility (45), cell division (46), secondary metabolite production (47), and various transport and metabolic functions (48). How the CheA1 and CheA4 proteins could have opposite effects on the transcription and abundance of RpoN is not yet known. A unique feature of RpoN-dependent transcription is the requirement of an activator to stimulate the σ^54^-RNA polymerase holoenzyme (σ^54^-holoenzyme) to form a complex able to initiate transcription (49). NtrC is an enhancer-binding protein that, in its phosphorylated form, is required for RpoN-dependent gene expression involved in nitrogen metabolism, and several non-nitrogen metabolisms regulated functions (37). NtrC was less abundant in the proteomes of all three mutants, and thus it is unlikely that NtrC alone is responsible for the changes observed in the chemotaxis mutants. Consistent with this hypothesis, NtrC also regulates the transcription of NifA in *A. brasilense* (34), and our data show that *nifA* promoter activity changed depending on the chemotaxis mutant considered and the availability of fixed nitrogen. The effect of CheA1 and CheA4 on the abundance of RpoN is thus likely dependent on unidentified enhancer-binding proteins and/or additional regulators. How CheA1 and CheA4 would alter the transcription of *rpoN* (and of *nifA*) or the activity of other regulators that alter physiology in the chemotaxis mutants is unknown. The signaling output of chemotaxis in bacteria, including *A. brasilense* (11, 50), results in changes in the activity of CheY response regulators that ultimately bind to flagellar motors to alter their rotation by protein-protein interactions (4, 51). Chemotaxis proteins are also known to interact with nonchemotaxis proteins, including Par-like proteins (52, 53) and phosphoenolpyruvate phosphotransferase systems in E. coli (51, 54, 55). It is thus possible that the effects of CheA1 and CheA4 on metabolism and physiology identified here depend on interaction with nonchemotaxis proteins which may themselves affect the activity of regulator(s). A third and nonexclusive possibility may be that CheA1 and CheA4 affect intracellular signaling. Chemoreceptors activity, and thus chemotaxis signaling, depends on *S*-adenosylmethionine and c-di-GMP in *A. brasilense* (25, 40, 56), which may be affected in the chemotaxis mutants. Regardless of the specific mechanisms, our data clearly point to previously unanticipated and complex functional links between chemotaxis and metabolism in *A. brasilense*.

## MATERIALS AND METHODS

### Strains, media, and growth conditions.

The bacterial strains used in this study are listed in Table 3. *A. brasilense* strain Sp7 (ATCC 29145) and mutant derivatives were grown at 28°C on minimal medium for *A. brasilense* (MMAB) supplemented with malate (10 mM [final concentration]) and 18.8 mM ammonium chloride (57). Cells were incubated with shaking (200 rpm) at 28°C. The *ΔcheA1*, *ΔcheA4*, and *ΔcheA1 ΔcheA4* mutants were described previously (Table 2). The growth of *A. brasilense* on different nitrogen sources was tested on solid MMAB medium supplemented with 18.8 mM NH_4_Cl or KNO_3_. Conjugation was performed on d-plates (8 g liter^−1^ Bacto Nutrient broth, 0.25 g liter^−1^ MgSO_4_ · 7H_2_O, 1.0 g liter^−1^ KCl, 0.01 g liter^−1^ MnCl_2_, 2% agar) and, after conjugation, MMAB with appropriate antibiotics was used for selection of *A. brasilense* transconjugants. Antibiotics were used at the following concentrations unless stated otherwise: tetracycline at 10 μg ml^–1^, ampicillin at 200 μg ml^–1^, kanamycin at 30 μg ml^–1^, and gentamicin at 20 μg ml^–1^. The nitrogen-free medium used in the nitrogen fixation growth conditions was MMAB without added NH_4_Cl. For determining growth rate and latent-phase times, strains were grown in 5 ml of MMAB at 28°C for 16 h, diluted to an optical density at 600 nm (OD_600_) of 0.1, and grown in MMAB for 18 h in microplates. Growth was measured overtime using a 800 TS absorbance reader with Gen5 software (Bio-Tek Instruments, Winooski, VT). Measurements of cultures at OD_600_ were made every 20 min. The lag phase (the delay in cell growth) of the bacterial cultures was calculated manually. For the swim assay, a single colony from each strain was inoculated in MMAB and grown until the OD_600_ was 0.8. The culture was then washed once with Che buffer, and 5 ml of the culture was placed on top of the MMAB with 0.3% agar. The plate was incubated at 28°C for 96 h, and the ring diameter was measured.

**TABLE 3:** Strains and plasmids used in this study

Strain or plasmid	Description	Reference or source
Strains		
*A. brasilense*		
Sp7	Wild-type strain	ATCC 29145
*ΔcheA1*	Δ*cheA1*::*gusA-*Km in Sp7 (Km^r^)	7
*ΔcheA4*	Δ*cheA4*::Gm in Sp7 (Gm^r^)	9
*ΔcheA1 ΔcheA4*	Δ*cheA1*::*gusA-*Km Δ*cheA4*::Gm in Sp7 (Km^r^, Gm^r^)	13
*rpoN:*:Km^r^	*rpoN:*:Km in Sp7 (Km^r^)	31
*E. coli*	General cloning: F^–^ *mcrA* Δ(*mrr-hsdRMS-mcrBC*) ϕ80*lacZ*ΔM15 Δ*lacX74 recA1 araD139* Δ(*ara leu*)*7697 galU galK rpsL* (StrR) *endA1 nupG*	Invitrogen
		
Plasmids		
pRK2013	Helper plasmid for triparental mating (ColE1 replicon, Tra, Km^r^)	71
pBBR-MCS3	Broad-host-range vector (Tc^r^)	70
pBBRCheA1	pBBR-MCS3 containing *cheA1* (Tc^r^)	8
pBBRCheA1ΔTMX	pBBR-MCS3 containing *cheA1*ΔTMX (Tc^r^)	8
pFUS	Broad-host-range vector with promoterless *gusA* (Tc^r^)	59
pFUSP*_rpoN_*	pFUS vector containing P*_rpoN_* (Tc^r^)	This study
pFUSP*_nifA_*	pFUS vector containing P*_nifA_* (Tc^r^)	This study
pFUSP*_nifH_*	pFUS vector containing P*_nifH_* (Tc^r^)	This study
pRK415	Broad-host-range vector (Tc^r^)	72
pRK415CheA4	pRK415 containing *cheA4* (Tc^r^)	9

### Nitrogen source utilization assays.

To assess cell growth in the presence of potassium nitrate, cells were grown in MMAB to the midexponential phase (OD_600_ = 0.8), washed three times with chemotaxis buffer (10 mM phosphate buffer [pH 7.0], 1 mM EDTA) (56), and rediluted to an OD_600_ of 0.1 in 1 MMAB with 20 mM KNO_3_. Cells were grown with the constant shaking at 28°C for 20 h. Measurements of cultures at OD_600_ were made every 20 min. The specific growth rate (μ) was calculated from five consecutive OD_600_ measurements (μ = Δln OD_600_/Δ*t*, where *t* is time). To assess cell growth under nitrogen fixation conditions, cells were prepared as described above for the experiment with potassium nitrate. Cultures were normalized to an OD_600_ of 0.2, and 50 μl was plated on MMAB made with Noble agar with or without NH_4_Cl. Spots were dried on the bench to ensure liquid evaporation and then incubated for 96 h at 28°C. The spots were cut out using the wide ends of 1,000-μl tips and placed in 1 ml of chemotaxis buffer. The cultures with agar cutouts were sonicated for 5 s at 5 W with a cell dismembrator (model 100; Fisher Scientific, Waltham, MA) to ensure cell detachment from the agar slice. The cultures were serially diluted and plated onto TY medium (tryptone, 10 g liter^−1^; yeast extract, 5 g liter^−1^; agar, 15 g liter^−1^) to ensure cell recovery after nitrogen limitation. The plates were incubated for 48 h at 28°C, and the CFU were counted. The survival of the strains was calculated as the ratio of CFU plated from nitrogen-free medium versus CFU plated from NH_4_Cl medium. A colorimetric assay to detect nitrogen fixation was performed as follows. The cells were grown for 16 h at 28°C in a shaker incubator and then washed three times with a chemotaxis buffer. Next, the OD_600_ was adjusted to 1.0, and the cells were concentrated 10-fold. Portions (30 μl) of the culture were injected into 3 ml of nitrogen-free semisolid MMAB made with 0.3% Noble agar and supplemented with 62.5 ml of 0.04% solution of bromothymol blue (Sigma, St. Louis, MO). Tubes were incubated for 2 days at 28°C and 3 more days at room temperature.

### DNA manipulations, cloning, and sequence analysis.

General cloning techniques were used as described previously (58). *A. brasilense* genomic DNA was isolated using a kit according to the manufacturer’s manual (Promega, Madison, WI). To construct plasmids carrying putative promoter regions of *rpoN*, a DNA region upstream of the putative translation start and encompassing ∼600 bp was amplified and cloned into a XhoI-HindIII region of digested pFUS vector (59) using the primer pair EG115 (F *rpoN*) (5′-TTAAAGCTTTCTTCCGCGGCATGTCGGTGG-3′)/EG116 (R *rpoN*) (5′-CATCTCGAGGGGCAGGGAAACCGCACTAGA-3′). Putative promoter regions of *nifA* and *nifH* were amplified and inserted into a XhoI-EcoRI region of digested pFUS using the primer pairs EG98 (F *nifA*) (5′-AAACTCGAGCAGACGCTCGGCCATGTCCAG-3′)/EG99 (R *nifA*) (5′-TTTGAATTCTGACCTCATAGATGGTCAGCA-3′) and EG94 (F *nifH*) (5′-AAACTCGAGCCAACGGCGGCGACCTCGACG-3′)/EG95 (R *nifH*) (5′-TTTGAATTCTGGCGCAAAGACATGGGAGGT-3′). The primers were designed to clone the promoter region at the 5′ end of the promoterless reporter gene (*gusA*) present on pFUS. Treatment of DNA with restriction enzymes was performed in accordance with the manufacturer’s specifications (New England Biolabs, Ipswich, MA). DNA sequencing was performed at the Sanger Sequencing Core Facility (University of Tennessee, Knoxville, TN). Sequence analysis to verify all constructs was performed using BLAST (https://blast.ncbi.nlm.nih.gov/Blast.cgi).

### **β**-Glucuronidase activity assay.

The β-glucuronidase activity was determined quantitatively using the substrate *p*-nitrophenyl-β-d-glucuronide, as described previously (60), with some modifications. Each culture was assayed in triplicate in three independent experiments. To test promoter activity, a fluorescence assay for the *gusA* gene in *A. brasilense* cells carrying pFUS or pFUSP*_rpoN_* was performed using a high-throughput β-glucuronidase assay performed in microplates. Briefly, *A. brasilense* strains were grown overnight in MMAB with appropriate antibiotics, washed with 0.9% NaCl, and resuspended to an OD_600_ of 0.4 in 0.9% NaCl. Portions (125 μl) of the cultures were mixed with 500 mM β-glucuronidase buffer (50 mM NaPO_4_ [pH 7.0], 10 mM β-mercaptoethanol, l0 mM EDTA, 0.1% sodium lauryl sulfate, 0.1% Triton X-100) in 1.5-ml tubes (USA Scientific), and 100 mM toluene was added to ensure cell lysis (61). The samples were incubated for 30 min at 37°C, followed by the tubes being left open under a chemical hood to ensure partial evaporation of toluene, before the tubes were moved to 28°C for 5 min. Then, 200-ml portions of the solutions of lysed cells were aliquoted into the 96-well flat-bottom plates, and 5 μl of *p*-nitrophenyl-β-d-glucuronide (ACROS Organics) at 35 mg ml^−1^ was added to each well and mixed five times by pipetting. Reaction measurements were taken every 5 min for 1 h and 30 min using an 800 TS absorbance reader with Gen5 software with a 405-nm filter (Bio-Tek Instruments). To study the activity of the *rpoN*, *nifH*, and *nifA* promoters under nitrogen-fixing conditions, the cultures were grown overnight in the MMAB with nitrogen and carbon source supplemented with tetracycline. Cultures were washed three times with a chemotaxis buffer and reinoculated into MMAB without nitrogen source at an OD_600_ of 0.5. Bacteria were incubated at 28°C for 24 h, and p-nitrophenyl-β-d-glucuronide (ACROS Organics) was added to each well, followed by mixing five times by pipetting. Reaction measurements were taken every 2 min for 1 h using an 800 TS absorbance reader with Gen5 software.

### Sample preparation for LC-MS/MS.

Sample preparation for liquid chromatography-tandem mass spectrometry (LC-MS/MS) was performed as follows. Strains were grown on MMAB plates with corresponding antibiotics, and 5 ml of liquid MMAB was inoculated and incubated at 28°C for 16 h. Next, 5-ml cultures were inoculated into fresh tubes containing 45 ml of MMAB, followed by incubation at 28°C for 16 h. The samples were then reinoculated into flasks containing 500 ml of fresh MMAB, followed by incubation at 28°C until reaching an OD_600_ of 0.5. The cells were spun down in sterile Sorvall tubes at 6,000 rpm in Sorvall centrifuge for 15 min. The pellets were washed three times with a chemotaxis buffer and stored at –80°C. Three biological replicates for each strain were prepared for LC-MS analysis. The experimental design scheme is presented in Fig. S1. The β-glucuronidase activity was measured as described previously.

### Protein extraction and digestion.

Cell pellets were suspended in SDS lysis buffer (2% in 100 mM NH_4_HCO_3_ and 10 mM dithiothreitol). The samples were physically disrupted by bead beating (0.15 mm) at 8,000 rpm for 5 min. Crude lysates were boiled 5 min at 90°C. Cysteines were blocked by adjusting each sample to 30 mM iodoacetamide, followed by incubation in the dark for 15 min at room temperature. Proteins were precipitated using a chloroform-methanol-water extraction (62). Dried protein pellets were resuspended in 2% SDS (100 mM NH_4_HCO_3_), and protein amounts were estimated by performing a bicinchoninic acid (BCA) assay. For each sample, an aliquot of ∼500 μg of protein was digested using two aliquots of sequencing-grade trypsin (Promega, 1:75 [wt/wt]) twice overnight, followed by a 3-h incubation at 37°C. The peptide mixture was adjusted to 0.5% formaldehyde to precipitate SDS. Hydrated ethyl acetate was added to each sample at a 1:1 (vol/vol) ratio three times to effectively remove the SDS. Samples were then placed in a SpeedVac concentrator (Thermo Fisher Scientific) to remove the ethyl acetate and further concentrate the sample. The peptide-enriched flow through was quantified by a BCA assay, desalted on RP-C18 stage tips (Pierce Biotechnology), and then stored at −80°C.

### Protein identification and quantitation.

All samples were analyzed on a QExactive Plus mass spectrometer (Thermo Fisher Scientific) coupled with a Proxeon EASY-nLC 1200 liquid chromatography (LC) pump (Thermo Fisher Scientific). Peptides were separated on a 75-μm-inner-diameter microcapillary column packed with 25 cm of Kinetex C_18_ resin (1.7 μm, 100 Å; Phenomenex). For each sample, a 2-μg aliquot was loaded in buffer A (0.1% formic acid, 2% acetonitrile) and eluted with a linear 150-min gradient of 2 to 20% of buffer B (0.1% formic acid, 80% acetonitrile), followed by an increase in buffer B to 30% for 10 min, another increase to 50% buffer for 10 min, and concluding with a 10-min wash at 98% buffer A. The flow rate was kept at 200 nl/min. MS data were acquired using Thermo Xcalibur software v4.27.19, a topN method where N could be up to 10. Target values for the full scan MS spectra were 1 × 10^6^ charges in the 300- to 1,500-*m/z* range, with a maximum injection time of 25 ms. Transient times corresponding to a resolution of 70,000 at *m/z* 200 were chosen. A 1.6-*m/z* isolation window and fragmentation of precursor ions were performed by using higher-energy C-trap dissociation with a normalized collision energy of 30 eV. MS/MS scans were performed at a resolution of 17,500 at *m/z* 200 with an ion target value of 1 × 10^6^ and a maximum injection time of 50 ms. Dynamic exclusion was set to 45 s to avoid repeated sequencing of peptides.

### Proteome data analysis.

MS raw data files were searched against the *A. brasilense* Sp7 proteome database, to which common contaminating proteins had been added. A decoy database, consisting of the reversed sequences of the target database, was appended in order to discern the false discovery rate at the spectral level. For standard database searching, the peptide fragmentation spectra (MS/MS) were analyzed by Proteome Discoverer v2.2. The MS/MS data were searched using MS Amanda v2.0 (63) and were configured to derive fully tryptic peptides with settings for high-high MS/MS data: an MS1 mass tolerance of 5 ppm and an MS2 mass tolerance of 0.02 Da. A static modification on cysteines (iodoacetamide, +57.0214 Da), a dynamic modification on methionine (oxidation, 15.9949), and aspartate and glutamate (methylation, 14.016) were considered. The results were processed by using Percolator (64) to estimate *q* values. Peptide spectrum matches and peptides were considered identified at a *q* value of <0.01. For label-free quantification, MS1-level precursor intensities (area) were derived from the Minora Feature and Precursor ion quantifier nodes using default parameters. Missing values were imputed (according to the low-abundance resampling method), and proteins were normalized by the total peptide amount using Proteome Discoverer. To test for differential protein abundances for all comparisons, the *P* value/group ratio was calculated by three-way analysis of variance (ANOVA) and Tukey’s HSD *post hoc* tests. The *P* values were then subjected to Bonferroni’s correction method, with a *P* value threshold of 0.05 to eliminate false-positive protein changes. Differentially expressed proteins are defined as having >2-fold changes compared to the reference abundance (absolute value log_2_-fold change of >1) and a *P* value of <0.05. Data set preparations and statistical analysis were performed using customized Python algorithms. A sample code is provided (https://github.com/lvo5/Alexandre_lab_omicscode).

### COG analysis.

To map COG annotations with respective protein IDs, a fasta file of the whole proteome in *A. brasilense* Sp7 was acquired using UniProt. The fasta file of the whole proteome was subsequently submitted to WebMGA (http://weizhong-lab.ucsd.edu/webMGA/server/cog/), an open-source web service for metagenomic analysis (65). Specifically, the sequences of each protein were mapped with COG IDs by predicting the relatedness to protein sequences presented in the COG database. A cutoff prediction of 0.05 was used for the mapping algorithm. A total of 5,029 proteins in the proteome were mapped to respective COG IDs, and 608 proteins were not mapped. Mapped COG IDs were functionally annotated using the updated 2014 version of the 2003 COG annotation files (https://www.ncbi.nlm.nih.gov/COG/) (66). In brief, each COG ID provided by the WebMGA algorithm was mapped with the respective functional categories provided by the listcogs.txt files in the 2003 COG file in the COG database. For proteins that have multiple COG IDs, the functional categories were merged, and repeated classifications were manually removed. General analyses were performed using a customized Python algorithm. The unmapped proteins or proteins that have three empty biological replicate abundance values were not considered for any COG analyses. To determine COG ID distribution, the numbers of each functional category were counted and normalized to the total numbers of functional categories presented in the *A. brasilense* Sp7 proteome. To determine the proteome abundance distribution based on functional categories, the total averaged protein abundances of each functional category were summed and normalized to the total number of protein abundance for wild-type Sp7 and mutants. A Fisher exact test was used to determine whether a particular functional category is enriched in a gene set compared to the whole proteome. In brief, the number of differentially expressed or not differentially expressed proteins in or not in a functional category were set up in contingency tables for both downregulated and upregulated gene sets for all comparisons. The tables were then subjected to a hypergeometric function to provide *P* values. All of the functions are provided in the *scipy* package in Python (67). The full code is provided elsewhere (https://github.com/lvo5/Alexandre_lab_omicscode/tree/master/cog_analysis_proteome).

### Hierarchical clustering analysis.

For the clustering analysis, log_2_-fold changes for all replicates for all comparisons were averaged. The values were then subjected to hierarchical clustering using a customized Python code with a statistical package called *scipy*. The clustering was done using Ward’s method with a distance threshold criterion of 10. In the hierarchical clustering analysis, the distance threshold of 10 provided the most optimized clustering of patterns based on the manual visualization of a log_2_-fold change expression pattern distribution within clusters. In brief, each expression profile for proteins for all comparisons are subjected to minimum variance criterion; thus, proteins with similar expression profiles or with minimal variance compared to the expression profile of neighboring proteins were considered to be a cluster. Ten unique clusters were formed using the described threshold conditions. Proteins within each cluster were subjected to COG annotation and classification, and the COG-term distribution for each cluster was determined as described above. The code is available at https://github.com/lvo5/Alexandre_lab_omicscode/tree/master/pattern_clustering_proteome (67).

### Metabolomics.

Sample preparation for LC-MS metabolomics was performed as follows. Strains were grown on MMAB plates with corresponding antibiotics, and single colonies were used to inoculate 5 ml of liquid MMAB that was then incubated at 28°C for 16 h. Next, 1-ml portions of these cultures were reinoculated into fresh 9 ml of MMAB, followed by incubation at 28°C until the sample reached an OD_600_ of 0.8. Cells were spun down in sterile culture tubes at 3,500 rpm in a Sorvall centrifuge for 15 min. Pellets were stored at –80°C for further analysis. Three biological replicas (for Sp7, *ΔcheA1*, and *ΔcheA4* strains) and two biological replicas for the *ΔcheA1 ΔcheA4* mutant (due to technical difficulties) of each strain were prepared for LC-MS analysis. Samples were separated on a Phenomenex Synergi Hydro RP column (2.5 μm, 100 mm × 2.0 mm) as reported previously (68, 69). The column was kept at 25°C. The mobile phases used to elute the metabolites were 97:3 water-methanol with 11 mM tributhylamine and 15 mM acetic acid (phase A) and 100% methanol (phase B). The multistep gradient for the 25-min method used a flow rate of 0.2 ml min^−1^. The Thermo Scientific Exactive Plus Orbitrap mass spectrometer used an electrospray ionization probe operating in negative mode. The scan range was 72 to 1,000 *m/z*. To test for differential metabolites abundances for all comparisons in metabolomics data set, the *P* value/group ratio was calculated by three-way ANOVA and Tukey’s HSD *post hoc* tests. No *P* value correction method was applied. Differentially expressed metabolites are defined as having nonzero fold changes compared to the reference abundance (an absolute value of log_2_-fold change of >0) and a *P* value of <0.05. Data set preparations and statistical analysis were performed using customized Python algorithms, as described above.

### Statistical analysis.

To compare wild-type and mutant phenotypes (swim assay, growth rate, CFU counts, and promoter activity), we determined average values from at least three independent experiments and performed pairwise two-sample *t* tests assuming equal variances (alpha level, 0.05) using Prism (v8) software (GraphPad Software, Inc., San Diego, CA). For principal-component analysis (PCA) of differentially expressed proteins set in proteomics and metabolomics data sets, log_2_-fold changes of all differentially expressed proteins or metabolites were transformed into two-dimensional (2D) principal components and standardized to both principal component values of 0. Each category of principal components represents the group of comparisons for three replicates. To model 95% confidence intervals of principal components as ellipses, covariance matrices of the principal components for all three replicates for each comparison were determined. The centers of mass of the ellipses were determined by averaging the principal components for all three replicates for each comparison. The angles of rotation of the ellipses were determined by the inverse tangents of the normalized covariance matrices between both principal components. The lengths of minor axes and major axes of the ellipses were modeled using the following equations:

(1) $$L_{\text{minor}}=2\times {\text{SEM}}_{\mathrm{PC}1}\times f\left(x=\left(1\;+\;c\right)/2,v=n\;-\;1\right)$$

(2) $$L_{\text{major}}=2\times {\text{SEM}}_{\mathrm{PC}2}\times f\left(x=\left(1\;+\;c\right)/2,v=n\;-\;1\right)$$

SEM*_PC_*_1_ or SEM*_PC_*_2_ is the standard error of mean of principal component 1 and 2, respectively. *f*(*x*, *v*) represents the percent point function, where *x* is a real number and *v* is a degree of freedom. *c* represents the confidence, which is 0.95. *n* represents the number of samples or replicates, which is 3. The sample code for PCA analysis is provided online (https://github.com/lvo5/Alexandre_lab_omicscode/blob/master/PCA_analysis_github/PCAanalysis.ipynb). For the correlation analysis, log_2_ abundances in all samples were averaged, and proteins with more than two missing abundance data were eliminated from the analysis. A Pearson’s correlation test was performed on the data set with averaged log_2_ abundances of all samples to obtain the value of the coefficient of correlation, *r*^2^ (70).

### Data availability.

All proteomics MS data in this study were deposited at the MASSIVE repository (https://massive.ucsd.edu/). The project identifier is MassIVE MSV000086008.
