# Supplementary figures and images for: The Azospirillum brasilense Core Chemotaxis Proteins CheA1 and CheA4 Link Chemotaxis Signaling with Nitrogen Metabolism
Source: mSystems. 2021 Feb 16;6(1):e01354-20. doi: 10.1128/mSystems.01354-20 (PMC8561660; doi:10.1128/mSystems.01354-20)

a.

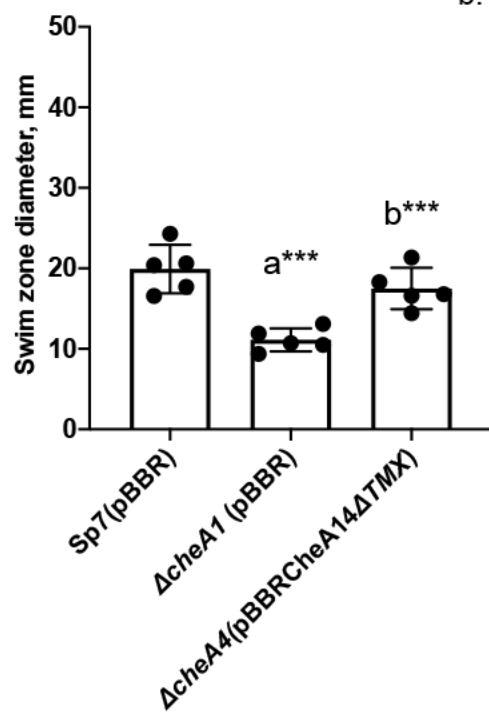

b.

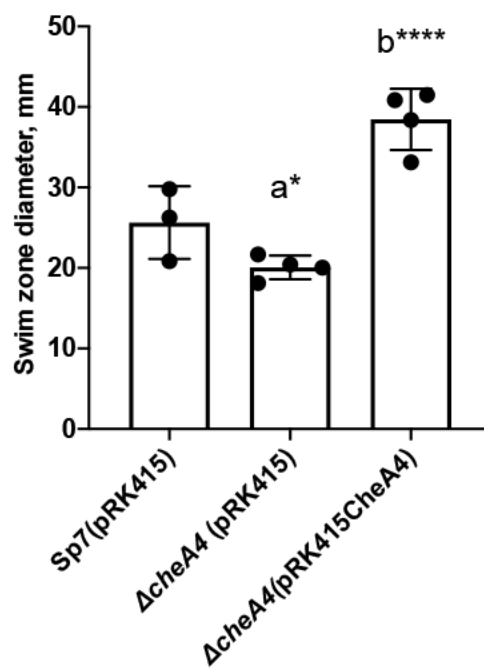

Supplement: FIG S1 [file msystems.01354-20-sf001.pdf]
